# Tumor- and circulating-free DNA methylation identifies clinically relevant small cell lung cancer subtypes

**DOI:** 10.1016/j.ccell.2024.01.001

**Published:** 2024-01-25

**Authors:** Simon Heeke, Carl M. Gay, Marcos R. Estecio, Hai Tran, Benjamin B. Morris, Bingnan Zhang, Ximing Tang, Maria Gabriela Raso, Pedro Rocha, Siqi Lai, Edurne Arriola, Paul Hofman, Veronique Hofman, Prasad Kopparapu, Christine M. Lovly, Kyle Concannon, Luana Guimaraes De Sousa, Whitney Elisabeth Lewis, Kimie Kondo, Xin Hu, Azusa Tanimoto, Natalie I. Vokes, Monique B. Nilsson, Allison Stewart, Maarten Jansen, Ildikó Horváth, Mina Gaga, Vasileios Panagoulias, Yael Raviv, Danny Frumkin, Adam Wasserstrom, Aharona Shuali, Catherine A Schnabel, Yuanxin Xi, Lixia Diao, Qi Wang, Jianjun Zhang, Peter Van Loo, Jing Wang, Ignacio I. Wistuba, Lauren A. Byers, John V. Heymach

**Affiliations:** 1 Department of Thoracic/Head & Neck Medical Oncology, the University of Texas MD Anderson Cancer Center, Houston, TX, USA; 2 Epigenetic and Molecular Carcinogenesis, The University of Texas MD Anderson Cancer Center, Houston, TX, USA; 3 Department of Translational Molecular Pathology, the University of Texas MD Anderson Cancer Center, Houston, TX, USA; 4 Medical Oncology Department, Hospital del Mar, Barcelona, Spain; 5 Department of Genetics, The University of Texas MD Anderson Cancer Center, Houston, TX, USA; 6 Graduate School of Biomedical Sciences, The University of Texas MD Anderson Cancer Center UTHealth Houston, Houston, TX, USA; 7 Laboratory of Clinical and Experimental Pathology, IHU RespirERA, Nice Hospital, University Côte d’Azur, Nice, France; 8 Department of Medicine, Division of Hematology and Oncology, Vanderbilt University Medical Center, Nashville, TN, USA; 9 Department of Genomic Medicine, The University of Texas MD Anderson Cancer Center, Houston, TX, USA; 10 Pulmonary Dept, Ziekenhuisgroep Twente, Hengelo, The Netherlands; 11 National Korányi Institute of Pulmonology, Budapest, Hungary; 12 7th Respiratory Medicine Dept, Athens Chest Hospital, Athens, Greece; 13 2nd Respiratory Medicine Dept, Athens Chest Hospital, Athens, Greece; 14 Dept of Medicine, Pulmonology, Institute, Soroka Medical Center, Ben-Gurion University, Beer-Sheva, Israel; 15 Nucleix Ltd. Rehovot, Israel; 16 Nucleix Inc. San Diego, CA, USA; 17 Department of Bioinformatics and Computational Biology, the University of Texas MD Anderson Cancer Center, Houston, TX, USA; 18 The Francis Crick Institute, London, UK

## Abstract

Small-cell lung cancer (SCLC) is an aggressive malignancy composed of distinct transcriptional subtypes, but implementing subtyping in the clinic has remained challenging, particularly due to limited tissue availability. Given the known epigenetic regulation of critical SCLC transcriptional programs, we hypothesized that subtype-specific patterns of DNA methylation could be detected in tumor or blood from SCLC patients. Using genomic-wide reduced-representation bisulfite sequencing (RRBS) in two cohorts totallying 179 SCLC patients and using machine learning approaches, we report a highly accurate DNA methylation-based classifier (SCLC-DMC) that can distinguish SCLC subtypes. We further adjust the classifier for circulating-free DNA (cfDNA) to subtype SCLC from plasma. Using the cfDNA classifier (cfDMC) we demonstrate that SCLC phenotypes can evolve during disease progression, highlighting the need for longitudinal tracking of SCLC during clinical treatment. These data establish that tumor and cfDNA methylation can be used to identify SCLC subtypes and might guide precision SCLC therapy.

## Introduction

Small cell lung cancer (SCLC) is a highly aggressive form of lung cancer with limited treatment options and generally poor prognosis. SCLC patient outcomes are only modestly improved with the addition of immunotherapy to frontline platinum-etoposide chemotherapy in an unselected population ^1,2^. Currently, there are no targeted therapies or predictive biomarkers in routine clinical use for SCLC patients, although several are currently under investigation including DLL3 protein expression for the use of DLL3-directed CAR-T cell ^3^ or bispecific-antibody targeting ^4,5^ as well as SLFN11 expression to select patients for PARP-inhibitor treatments ^6^. Despite these efforts, the 2-year survival rate has not changed appreciably during the past decade ^7^.

Although SCLC has historically been treated as a single disease entity, recent studies have revealed that there are biologically distinct subgroups of SCLC, and that these subgroups have different therapeutic vulnerabilities and hence could be used for tailoring treatment regimens ^8–11^. We recently reported on four distinct SCLC subgroups based on mRNA profiling ^10^. Three of the four subtypes are enriched in the predominant expression of specific transcription factors, *ASCL1* (SCLC-A), *NEUROD1* (SCLC-N), and *POU2F3* (SCLC-P) while the fourth is an inflamed subtype (SCLC-I) associated with higher levels of PD-L1 and other checkpoint factors, and higher levels of interferon signaling and epithelial to mesenchymal transition (EMT) based on their transcriptomic signature ^10^. Importantly, in two independent analyses, the SCLC-I subtype is associated with the greatest benefit of the addition of immunotherapy to platinum-etoposide chemotherapy demonstrating the potentially predictive value of SCLC subtyping ^10,12^.

Given the growing recognition that SCLC is comprised of subtypes with distinct therapeutic vulnerabilities ^10,13,14^, the development of practical biomarkers for identifying patients likely to benefit from those therapies is urgently needed. Unfortunately, development of biomarkers in SCLC is hindered by the lack of access to tissue as diagnostic specimens are often limited to fine needle aspirations and surgery is rarely performed ^15^. Consequently, common subtyping approaches studied for SCLC - such as the use of mRNA expression signatures or multi-marker immunohistochemistry (IHC) - can typically be performed on only a subset of patients. These approaches also have shortcomings limiting their routine clinical adoption, such as mRNA degradation commonly seen in preserved SCLC specimens, and the use of subjective and time-intensive scoring methods used for multi-marker IHC assays.

In contrast to the tissue limitations, SCLC is often associated with a high shedding of circulating tumor DNA (ctDNA) and circulating tumor cells (CTCs) and consequently, liquid biopsy strategies have been extensively researched in this setting ^16–18^. While the development of circulating tumor cell derived xenograft models (CDX) allowed the mechanistic study of SCLC and has become indispensable for the development of novel therapeutic strategies ^19,20^, liquid biopsies are also used for the development of biomarker approaches. Recently, it has been shown that DNA methylation, as a surrogate to gene expression, can be used for the development of prognostic signatures as well as to differentiate *ASCL1*-dominant SCLC and *NEUROD1*-dominant SCLC from a third group of SCLC which is highlighted by the absence of *ASCL1* or *NEUROD1* dominance ^21^. These and other approaches, including profiling of plasma-derived nucleosomes ^22^ and fragmentomics analyses ^23^, have opened avenues for using liquid biopsies to guide precision medicine approaches in SCLC. However, previous analyses were limited by the absence of tumor specimens for direct comparison or profound subtyping of patients based on clinically validated gene expression-based subtyping which hampers the routine implementation of SCLC subtyping. Here, we therefore investigate the potential use of DNA methylation from both tumor and ctDNA in a cohort of 179 SCLC patients whose subtypes are assigned based on our recently established classification system ^10^. We develop machine learning approaches to allow the classification of SCLC subtypes using DNA methylation from both tissue and liquid biopsy samples in order to identify SCLC subgroups and enable precision medicine in SCLC.

## Results

### 1 Detection of SCLC using DNA methylation in plasma samples

**Figure 1:**
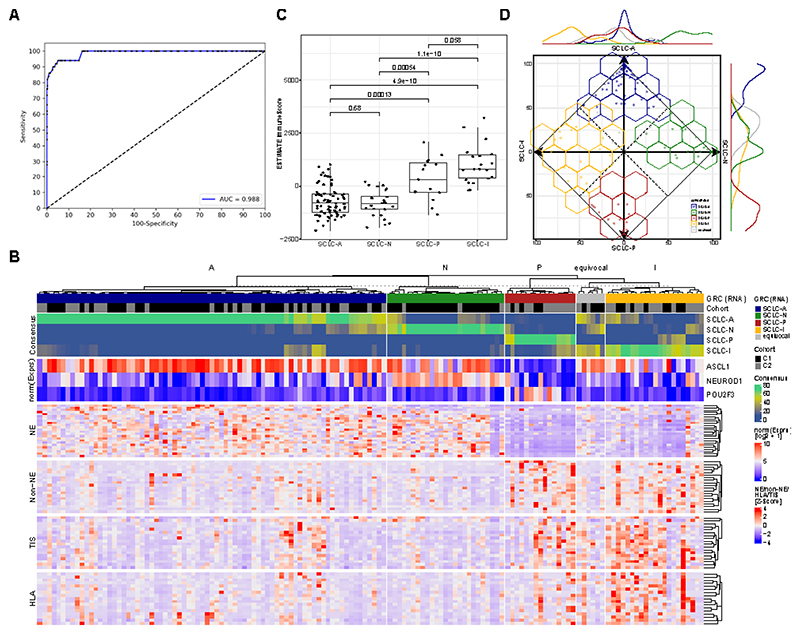
Detection and Classification of SCLC. **A** Receiver operator characteristics (ROC) analysis of a DNA methylation-based test for the detection of SCLC from plasma. **B** Predictive models were generated to classify SCLC based on RNA-seq (Gene Ratio Classifier; GRC) and consensus of several combined predictive models is shown. A subtype was called when the consensus > 0.5, else a sample was called equivocal. In addition, the expression of the three transcription factors ASCL1 (for SCLC-A), NEUROD1 (for SCLC-N) and POU2F3 (for SCLC-P) is shown normalized across the two cohorts. Furthermore, genes involved in neuroendocrine and non-neuroendocrine (Non-NE) as well as in tumor inflammation (TIS) and expression of HLA is shown. **C** Immune infiltration estimation using RNA-seq data (using the ESTIMATE algorithm). Boxplot shows the median as thick line, the box highlighting the first and third quartile with the whiskers highlighting 1.5x the interquartile range. **D** Characterization of SCLC consensus heterogeneity. The consensus agreement value for each subtype is plotted on the axis for each subtype by its consensus fraction of the respective subtype, demonstrating overlaps between SCLC subtypes. The line plot at the axis characterizes the distribution of subtypes across the axis. Wilcoxon test was used to compute p-values between groups. See also Figure S1, Table S1, and Data S1 and S2.

We hypothesized that DNA methylation can be used to detect SCLC in the circulation and to test this we initially utilized a methylation-sensitive digestion PCR assay designed previously to detect lung cancer (EpiCheck assay). Evaluation was based on a cohort of 52 SCLC cases of which 50 (17 Limited Stage SCLC (LS-SCLC) and 33 Extensive Stage SCLC (ES-SCLC)) passed quality control and 398 control cases (395 passed quality control) of which 137 cases have been used in an earlier validation study ^24^. The area under the curve for the detection was 0.988 (95% CI: 0.977-0.999; Figure 1A). Two different cut-offs were used for the detection, yielding a sensitivity and specificity of 100.0% (95% CI: 92.9%-100.0%) and 83.8% (95% CI: 79.8%-87.3%) with the low cut-off (EpiScore = 65) and 94.0% (95% CI: 83.5%-98.7%) and 94.9% (95% CI: 92.3%-96.9%) with the high cut-off (EpiScore = 74), respectively (Figure S1A), with high sensitivity in both LS-SCLC (Figure S1B) and ES-SCLC (Figure S1C). Four of the six markers used have also been assessed in the subtyping cohort (Table 1) with high methylation levels detected across all four subtypes (Table S1).

### 2 Cohort of clinical specimens for RNA-seq and DNA methylation profiling

Given our finding that DNA methylation was able to detect SCLC from plasma, we next hypothesized that DNA methylation can be exploited as a biomarker to subtype SCLC. To this end we investigated two independent cohorts of 105 and 74 samples respectively (Table 1). Generation of RNA-seq and RRBS data was feasible in both cohorts, though in C2, only RNA instead of tissue sections was provided for a subset of samples, leading to a lower number of samples with tissue methylation data due to the absence of DNA specimen in this subset. Reasons for unsuccessful analysis were low RNA or DNA content, low DV200 for RNA or unsuccessful library generation. Processed RNA-seq data is shown in Supplementary Data 1 (for cohort 1) and Supplementary Data 2 (cohort 2).

### 3 Clinical SCLC can be classified using a reduced machine learning RNA-seq signature

We previously reported that SCLC can be classified in four distinct subtypes using a gene expression classifier derived from non-negative matrix factorization (NMF) ^10^ and mRNA expression data from ^25^ both limited stage and ^1^ extensive stage SCLC specimens. However, our previously established NMF method is limited to analyzing cohorts only and thus we aimed to establish a predictive classifier to allow the subtyping of individual samples. Building on this analysis, we therefore developed a gene ratio classifier (SCLC-GRC) in order to reduce the number of genes required to subtype tumors and facilitate the subtype classification using different mRNA profiling methods. Using a consensus classification (see STAR methods) incorporating 181 genes, we were able to unambiguously classify the majority of samples into a single subtype, notably independent of the cohort and underlying RNA-seq method used (Table S1, Figure 1B). Across both cohorts, unambiguous subtyping was achieved for 136/142 (96%) of samples with RNA-seq data (Table S1). Classification was balanced across the four subtypes with 75/142 (53%), 25/142 (18%), 21/142 (15%), 15/142 (11%) representing the SCLC-A, SCLC-N, SCLC-P an SCLC-I subtypes, respectively. This distribution is comparable to the observed distribution in the IMpower133 study with SCLC-A - 51%, SCLC-N - 23%, SCLC-I – 18%, SCLC-P – 7% ^10^ (chi-sq *p* = 0.4186). Consistent with the prior reports of the four subgroups ^10^, the SCLC-A and SCLC-N samples in our cohort demonstrated a higher expression of neuroendocrine genes compared to SCLC-P and SCLC-I, while the SCLC-P and SCLC-I subgroups were characterized by a higher expression of HLA genes, tumor inflammation genes (TIS) (Figure 1B) as well as a higher percentage of tumor stroma and, hence, a lower percentage of tumor cells (Figure 1C) as calculated using RNA-seq deconvolution ^26^. Furthermore, using CIBERSORT deconvolution^27^, we identified increased immune cell infiltration in the SCLC-P and SCLC-I subtypes, respectively (Figure S1D. Importantly, the consensus of classification for each of the samples, retrieved from the overlap of 500 machine learning models highlighted certain distributions across the four subtypes, with samples acquiring properties of some of the other subtypes, suggesting that the SCLC-GRC approach is preserving information on the of intratumoral heterogeneity of SCLC subtype properties (Figure 1D). Only few specimens could not be classified (equivocal: 6/142; 4%) due to what appears to be technical limitations and RNA quality (Figure 1B). Consequently, with a success rate of 96%, our classification approach was highly accurate across different cohorts and RNA-seq technologies while comprised of a limited number of 181 genes. Thus, this assessment is technically less challenging than larger gene panels, and enables robust SCLC subtype classification from different cohorts and individual samples.

### 4 Genome-wide hypomethylation is characteristic of SCLC-P

**Figure 2:**
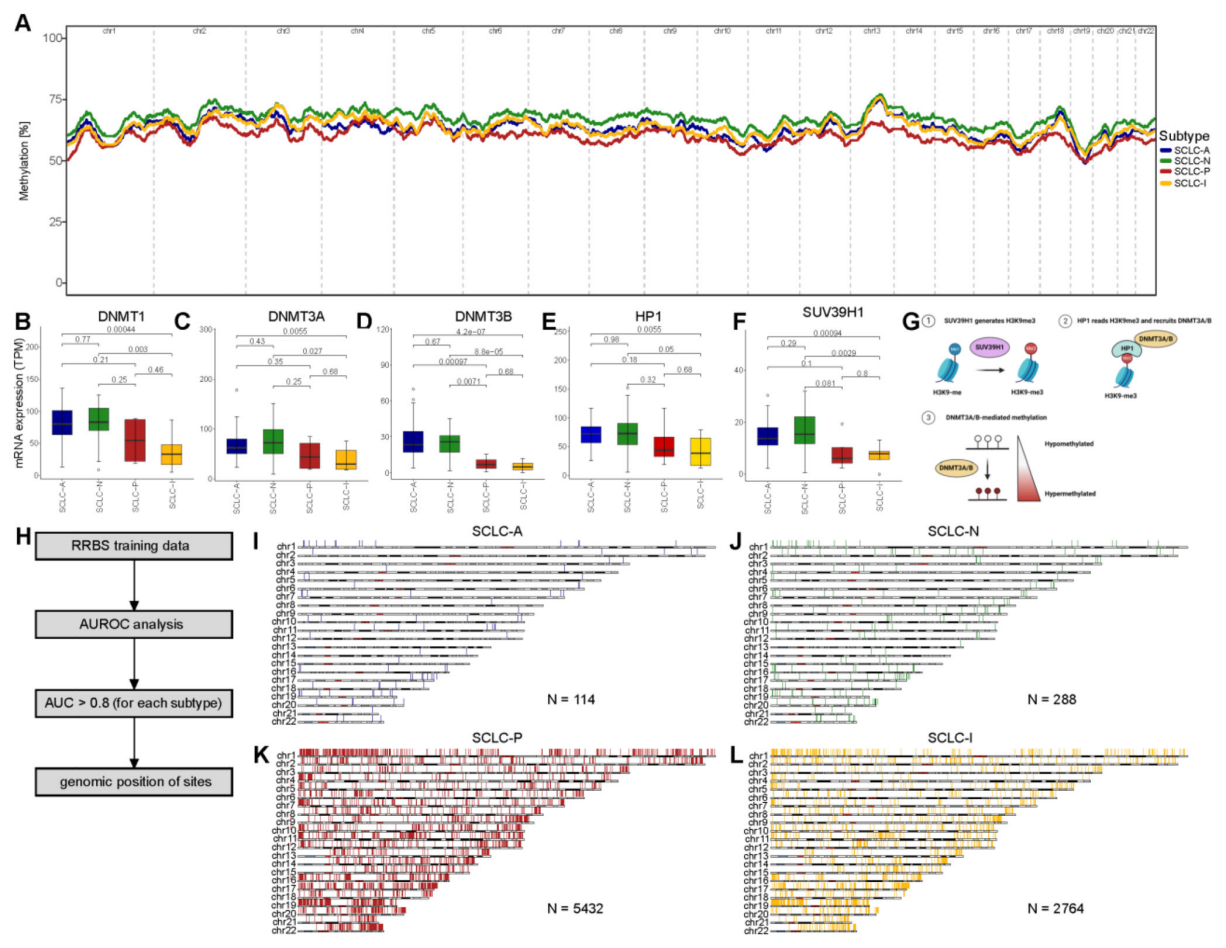
Subtype-specific DNA methylation in SCLC. **A** DNA methylation was assessed using reduced-representation bisulfite sequencing (RRBS) and DNA methylation was averaged per sample and subtype over 100kbp bins and the rolling average over 500 bins (= 50mbp) is highlighted in the c1 tumor samples. **B-G** Analysis of gene expression per SCLC subtype for DNA-methyltransferase 1 (DNMT1; **B**), DNA-methyltransferase 3A (DNMT3A; **C**) and 3B (DNMT3B; **D**), methionine adenosyltransferase 2A (MAT2A; **E**) and histone lysine methyltransferase (SUV39H1; **F**). **G** Overview of mechanism that links SUV39H1 expression with histone methylation. **H** Scheme highlighting the analysis and selection of DNA methylation sites associated with each of the SCLC subtypes using 100bp bins. By calculating the area under the curve by receiver operator characteristics (AUROC) we defined genomic region with high (AUC > 0.8) association with one the four respective subtypes. DNA methylation bins are shown related to their position within the genome for each chromosome for SCLC-A (**I**), SCLC-N (**J**), SCLC-P (**K**) and SCLC-I (**L**) and number of regions is stated for each subtype. Boxplot shows the median as thick line, the box highlighting the first and third quartile with the whiskers highlighting 1.5x the interquartile range. Wilcoxon test was used to compute p-values between groups. See also Figures S2-5 and Table S2.

We then analyzed the differences of genome-wide DNA methylation in our dataset. We averaged the methylation level across bins of 100kb width and calculated the mean for those bins per subtype. To determine the genome-wide methylation level, we calculated the rolling average over 500 bins (= 50Mbp). The analysis highlighted profound differences in the global methylation level per subtype, with the SCLC-P subtype presenting with a hypomethylated phenotype and SCLC-N with a hypermethylated phenotype, while SCLC-A and SCLC-I were comparable in cohort 1 (Figure 2A) as well as when filtering for tumor-intrinsic DNA methylation signals using the CAMDAC algorithm^28^ in a subset of samples in cohort 2 (Figure S2A; see STAR methods). The SCLC-P hypomethylation phenotype was also observed in cohort 2 while methylation patterns for the other subtypes appeared to differ between the cohorts (Figure S2B). We further analyzed 59 SCLC-derived cell lines across all four subtypes as well as two previously published datasets on cell lines. Interestingly, in cell lines, SCLC-P was hypermethylated (Figure S2C) contrary to tumor methylation analysis, which was confirmed in two independent datasets of cell lines from the NCI SCLC cell miner project ^29^ (Figure S2D) and the GDSC ^30^ (Figure S2E), highlighting limitations when working with cell line derived tumor methylation data.

To further explore these subtype-specific differences in global methylation, we analyzed expression of 73 genes responsible for reading, writing, or erasing DNA and histone methylation and found 47 (64%) of them to be significantly differentially regulated across subtypes (Table S2; Figure S3). In addition to the major DNA methyltransferases, *DNMT1* (Figure 2B), *DNMT3A* (Figure 2C) and *DNMT3B* (Figure 2D) and the S-Adenosylmethionine synthetase (*MAT2A*; Figure 2E) which creates S-adenosylmethionine (SAM) which is critical for methylation processes, we also found *SUV39H1* (Figure 2F) to be differentially expressed between the four SCLC subtypes, especially between neuroendocrine and non-neuroendocrine subtypes. *SUV39H1* is a methyltransferase that trimethylates histone H3 lysine 9 (H3K9) residues. Functionally, H3K9me3 recruits *HP1* and *DNMT3A*/*B* for stable methylation of DNA (Figure 2G)^31,32^, thereby linking histone methylation with induction of DNA methylation (Figures 2C, D, F, G). These data suggest that *SUV39H1*-*DNMT3A*/*B* axis is a candidate pathway contributing to differences in global methylation patterns across SCLC subtypes and highlighting further differences in epigenetic regulation of SCLC subtypes. Interestingly, the expression patterns of methylation effectors were distinct in SCLC cell line models, which might contribute to the discordance of global methylation patterns in cell lines compared to that in primary tumor samples (Figure S4; Table S2).

In order to further understand the genomic regions differing between SCLC subtypes, we analyzed the average methylation using bins of 100bp across the genome (Figure 2H). We utilized the training set of our combined RRBS data (see Materials and Methods) and used receiver-operator characteristics (ROC) to analyze the association of each 100bp bin with each of the four respective subtypes by computing the area under the curve (AUC) and filtered for highly associated sites with AUC > 0.8. We then highlight these highly associated sites according to their genomic location, for SCLC-A (Figure 2I), SCLC-N (Figure 2J), SCLC-P (Figure 2K) and SCLC-I (Figure 2L) as well as for each sample individually (Figure S5). Importantly, bins were spread across the different chromosomes confirming the genome-wide methylation differences.

### 5 DNA Methylation allows classification of SCLC specimens

**Figure 3:**
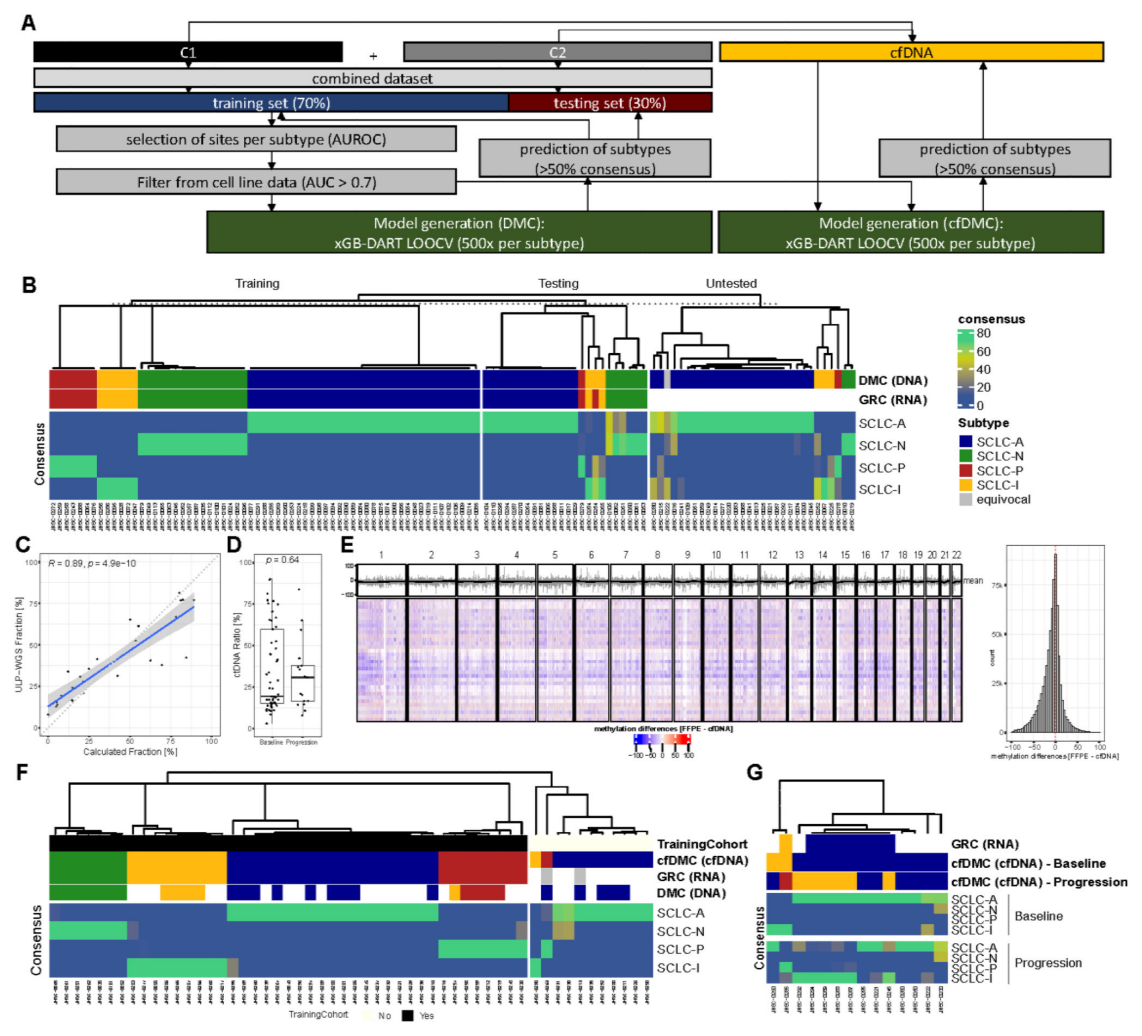
DNA methylation-based subtyping in SCLC. **A** Scheme describing the process to develop the SCLC DNA methylation classifier (SCLC-DMC). Both cohorts were combined and the dataset was split in a training and a testing set and highly predictive DNA methylation sites were selected using area under the receiver operator characteristics curve (AUROC) to create predictive models using extreme gradient boosting with Dropouts multiple Additive Regression Trees (xGB-DART) with leave one out cross validation (LOOCV). For each subtype, 500 models were individually trained. Performance was assessed on the testing set. A cfDNA adjusted consensus classification approach (SCLC-cfDMC) was created using the same DNA methylation sites as used for the SCLC-DMC to predict subtypes in liquid biopsies. **B** Classification of SCLC tissue specimen using the SCLC-DMC approach. Prediction of subtype is shown in the training set, the independent testing set as well as in samples were classification by RNA (GRC) was not possible due to the absence of RNA-seq data (untested). The consensus in percentage of agreement between the models is shown. **C** Correlation of computed circulating tumor DNA (ctDNA) fraction by ultra-low pass whole genome sequencing (ULP-WGS) and a classifier based on seven methylation sites (Calculated Fraction [%]). **D** Differences in ctDNA fraction per DNA methylation were compared between samples analyzed at baseline prior to treatment and samples at tumor progression. **E** Differences in genome-wide DNA methylation between tumor tissue samples and matched baseline plasma samples were compared. DNA methylation was averaged per sample and subtype over 100kbp bins and changes between tumor DNA methylation and plasma DNA methylation were analyzed for each 100kb bin for each patient represented by a row in the heatmap across each chromosome as highlighted above. Furthermore, mean methylation per bin across the samples is highlighted in grey color above the heatmap together with the rolling average depicted by a black line. A histogram to the right highlights the distribution of differences for each bin across all samples. **F** The classification of SCLC subtypes using the SCLC-cfDMA approach is shown in plasma sample taken at baseline prior to treatment. Additionally, to the consensus, the classification based on the gene-ratio approach (GRC) as well as based on the tissue DMC approach is shown. Samples with GRC classification were included in the training cohort and inclusion for each sample is shown. **G** Classification of SCLC-subtypes using the SCLC-cfDMC approach is shown for samples with matched baseline plasma and plasma at progression. Boxplot shows the median as thick line, the box highlighting the first and third quartile with the whiskers highlighting 1.5x the interquartile range. Wilcoxon test was used to compute p-values between groups. See also Figures S6-9 and Table S3.

Our findings suggested that differences in DNA methylation could be exploited for the generation of biomarkers that are able to differentiate SCLC subtypes. Therefore, we combined the DNA methylation data from both cohorts and randomly split the combined dataset in a training and an independent testing set (70% and 30% of samples, respectively). The training set was used for both marker selection and model training to ensure that the testing set could be used for independent validation. DNA methylation sites for training have been associated with each of the four subtypes in the training set using ROC (Figure 3A; Table S3). Despite marketed differences in DNA methylation compared to cell lines, we furthermore filtered for DNA regions which have also been associated with the four subtypes in cell lines (AUC > 0.7) to enable the model to train on tumor-intrinsic signals and avoid overfitting the model based on tumor-stroma derived methylation data, which we expect to be a larger contribution in the SCLC-P and SCLC-I subtype. We then selected the top DNA methylation sites for each of the four subtypes by differences in DNA methylation level and AUC, and created models that were trained by randomly selecting 10, 50, or 100 methylation sites per subtype, since this has been shown to provide sufficient information (Figure S6A). Furthermore, methylation sites selected are specific to SCLC compared to data obtained from lung adenocarcinoma and pre-neoplasia as well as non-cancer controls (Figure S6B) ^33^. Similar to our approach on RNA-seq data (Figure 1B), we used a threshold of >50% consensus across the models to call a subtype. We ultimately selected 50 methylation sites/subtype for our final predictive model, as this provided classification with high accuracy (Figure S7A). Accuracy for our DNA methylation classifier (SCLC-DMC) in the independent testing set was 95.8% (95% CI: 78.9% - 99.9%; Kappa = 0.9286). Importantly, the SCLC-DMC approach allowed the subtyping of 30 additional samples for which no RNA-seq data was available and thus RNA-based classification was impossible (Figure 3B; Table S3). Interestingly, heterogeneity from the consensus approach was reduced in the DMC approach, compared to our GRC approach (Figure S7B; Figure 1D). In order to validate the performance of the assay and to ensure that tumor-intrinsic features have been used for the training, we used the DMC approach to also predict subtypes in a set of cell lines that had been classified previously (Figure S7C).^10^ Our SCLC-DMC approach was also capable of classifying SCLC cell lines across all four subtypes with an accuracy of 96.6% (95% CI: 88.1 – 99.6).

### 6 DNA Methylation is preserved in ctDNA and can be used for classification of SCLC subtypes

Since DNA methylation is highly conserved in plasma, we hypothesized that DNA methylation can also serve as biomarker in SCLC liquid biopsies. First, we established a DNA methylation-based assessment of ctDNA to calculate the ctDNA burden. While the highly sensitive method for SCLC detection (Figure 1A) only allows the assessment of SCLC presence/absence, an additional method that allows the quantification of ctDNA fraction could potentially enable more insights on data derived from tumor and thus quality of classification. Indeed, we found multiple DNA methylation sites that correlate with ctDNA fraction based on ultra-low pass whole genome sequencing (ULP-WGS) and we selected seven methylation sites which are highly and linearly correlated to ctDNA fraction (Figure S8A). By calculating the mean for the seven selected sites, we established a convenient and easy method to assess ctDNA in SCLC with high correlation to ULP-WGS (*R* = 0.89; p < 0.0001; Figure 3C). We further analyzed how the ctDNA fraction differed between samples at baseline and progression, and observed no significant differences (Figure 3D). Consequently, samples selected at tumor progression yielded results comparable to samples at baseline, underscoring the applicability of our SCLC subtyping approach.

Based on the robust results from our tissue SCLC-DMC, we hypothesized that our approach could also be applied to SCLC plasma samples. Using a subset of five matched samples, we analyzed the differences between tumor DNA methylation and plasma DNA methylation and observed that SCLC DNA methylation patterns are indeed conserved in plasma (Figure 3E), enabling a liquid biopsy approach. We therefore utilized the same DNA methylation sites as selected for our SCLC-DMC for tissue samples, filtered for sites detected in plasma and refitted a model using only samples with GRC classification (*N* = 43/54 80%; SCLC-cfDMC; Figure 3A). Indeed, this allowed us to classify SCLC plasma samples with an accuracy of 100% (43/43) compared to the RNA-based SCLC-GRC and 93.3% (28/30) compared to the SCLC-DMC (Figure 3F; Table S3). Moreover, we observed excellent concordance with samples profiled only by our tissue-based SCLC-DMC to robustly detect all four SCLC subtypes from clinical plasma samples. Of note, all samples used for the classification were from untreated patients to allow correlation of subtypes with the associated tumor tissue. We also compared the DNA methylation levels selected for the training with DNA methylation data obtained from healthy donors^34^, and could demonstrate that baseline cfDNA samples from SCLC patients cluster generally distinctly to DNA methylation profiles from the healthy comparison (Figure S8B). Correlating global DNA methylation between healthy cfDNA and baseline samples, we observed a statistically significant drop in correlation for samples with higher ctDNA fraction (third and fourth quartile) compared to samples with lower ctDNA fraction (first and second quartile; Figure S8C).

Prior studies using single cell profiling from our group and others suggest that SCLC tumors can become more heterogenous, and shift their subtype, after progression on therapy. ^20,35,36^ To assess this, we analyzed a subset of patients, in which baseline samples as well as plasma sample at clinical progression were available. Our analysis of these samples demonstrated a strong heterogeneity in the sample subtype at progression as compared to their baseline classification (Figure 3G; Figure S9A). For example, in a large subset of patients, the SCLC subtype of their respective tumor switched from SCLC-A to SCLC-I at progression. Therefore, we further analyzed the promoter methylation levels in the cfDNA of patients with a baseline SCLC-A subtype whose did or did not demonstrate a subtype switch to SCLC-I. Indeed, in samples with subtype switching we saw marked differences in the promoter methylation of immune-related genes, such as *CXCL12 (T cell recruitment), CIITA (antigen presentation machinery transcription), STAT1 (inflammatory gene transcription)* as well as the interferon alpha and gamma receptors (*IFNRA1, IFNRA2, IFNGR1*) highlighting profound changes in the tumor:immune phenotypes (Figure S9B). Even though all those changes were not limited to the subtype switching samples this further highlights that analysis of promoter methylation from liquid biopsy samples can also provide information on tumor evolution under therapeutic pressure. Despite the switch to a more inflammatory phenotype, we did not detect any differences in PFS (HR = 0.49; 95% CI: 0.11 – 2.24) or OS (HR = 1.02; 95% CI: 0.27 – 3.9) for patients whose tumors switched to SCLC-I versus those that maintained SCLC-A subtype (Figure S9C). Treating SCLC cell lines with 2µM cisplatin for 9 days, did not alter DNA methylation in the respective genes, suggesting that the contribution of the tumor microenvironment might be required for subtype plasticity (Figure S9D).

### 7 DNA Methylation predicts drug response and clinical outcome similar to gene expression

**Figure 4:**
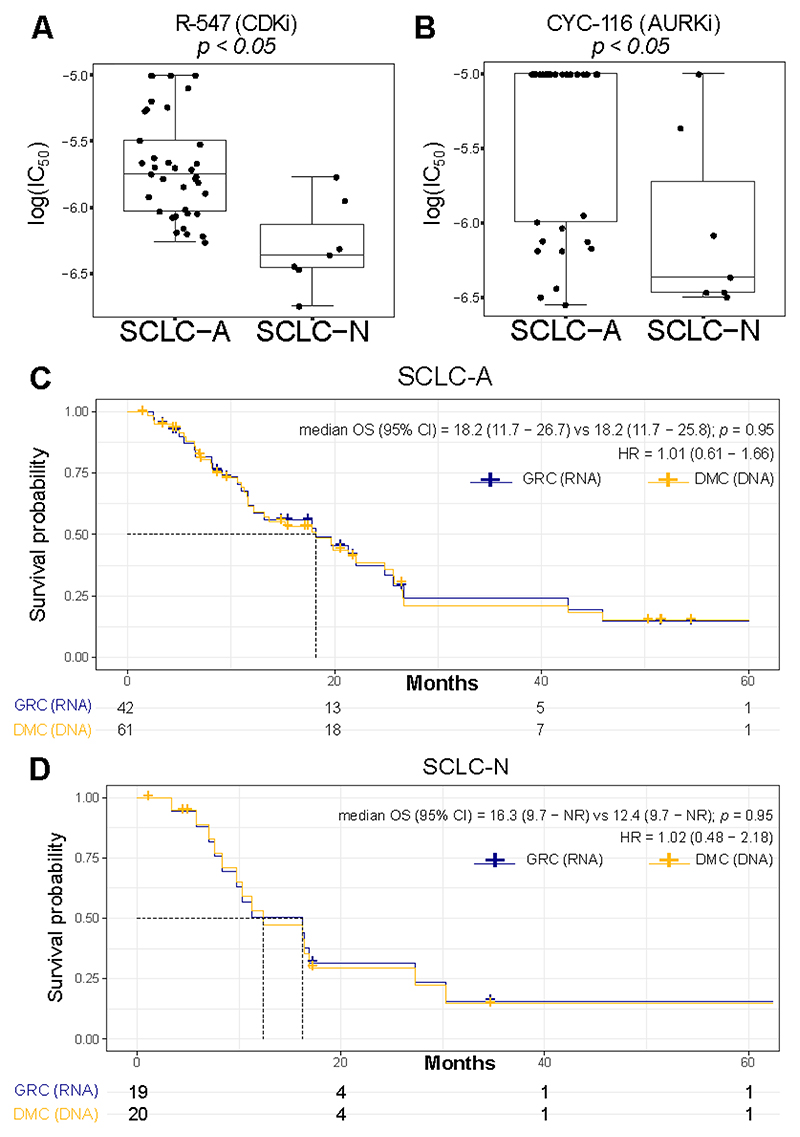
Influence of SCLC subtyping methods on in vitro drug screening and clinical outcome. Comparison of IC50 values for the **A** CDKi R-547 and the **B** AURKi CYC-116 between cell lines assigned to SCLC-A and SCLC-N using SCLC-DMC. **C-D** Clinical outcome depending on classification method used. Overall survival of SCLC patients stratified by classification using the SCLC-GRC (RNA-seq) and SCLC-DMC (DNA Methylation) method for **C** SCLC-A and **D** SCLC-N. Statistical significance is calculated using log-rank test. Cox-proportional hazard ratio is calculated and shown with 95% confidence interval. Boxplot shows the median as thick line, the box highlighting the first and third quartile with the whiskers highlighting 1.5x the interquartile range. Wilcoxon test was used to compute p-values between groups.

Previously, we demonstrated that, *in vitro*, cell lines assigned to SCLC-A and SCLC-N by gene expression possessed unique therapeutic vulnerabilities ^10^.To validate that these same vulnerabilities are preserved using the methylation classifier, we compared IC50 values for over 400 drugs ^37^ between methylation-assigned SCLC-A and SCLC-N subtypes and identified numerous distinct vulnerabilities between the groups. For example, as demonstrated with the gene expression classifier, SCLC-N cell lines were more sensitive to the CDK inhibitor (BCL2i) R-547 (Figure 4A), as well as to Aurora kinase inhibitor (AURKi). CYC-116 (Figure 4B). Collectively, these data provide evidence that DNA methylation is able to predict drug response *in* vitro similar to RNA-based classification.

Finally, to determine whether methylation- and RNA-based subtyping approaches yielded comparable clinical outcomes among SCLC patients, we used our SCLC-GRC or our SCLC-DMC for patients with known clinical outcomes. While many samples had both RNA and methylation data present, several of the patients were only subtyped by one of the two methods. To ensure adequate statistical power for the analysis, we focused on the two most prevalent subtypes, SCLC-A and SCLC-N, respectively. Importantly, when comparing the two approaches, overall survival was comparable for patients identified as SCLC-A (HR (95% CI) = 1.01 (0.61 – 1.66); Figure 4C) as well as for patients identified as SCLC-N (HR = 1.02 (0.48 − 2.18); Figure 4D) when using SCLC-GRC (RNA-seq) or SCLC-DMC (DNA methylation), demonstrating that DNA methylation and RNA-seq can be assessed and provide concordant results in the clinical setting.

## Discussion

Lung cancer histological subtypes are increasingly defined by transcriptomic features rather than solely by mutational signatures ^38^. This is especially true in small-cell lung cancer with its four distinct subtypes that are defined by specific gene expression rather than by targetable, or even distinct, genomic alterations. Indeed, advancement of personalized therapies in such a setting requires more complex clinical classification strategies. Consequently, we developed robust classifiers using gene expression data (SCLC-GRC) as well as DNA methylation (SCLC-DMC) to accurately and reliably predict SCLC subtypes in clinical specimens. Importantly, classification using SCLC-DMC was also established in plasma specimen addressing a critical need in SCLC, where tumor specimens are scarce and accurate liquid biopsy-based approaches are urgently needed. Both methods allow the precise classification of a transcriptionally defined tumor phenotype while the DNA methylation-based method allowed to further subtyping using liquid biopsy specimen. Consequently, DNA methylation-only strategies can be employed in settings where molecular analysis is performed primarily with DNA specimen, while the use of transcriptionally methods might enable the integration with additional signatures, for example for better description of the tumor microenvironment or assessment of marker genes ^39^.

To date, SCLC subtypes have been associated with the predominant expression of a transcription factor (ASCL-1, NEUROD1, or POU2F3) although it is worth noting that the SCLC-A, -N, -P, and -I subtypes were defined by clusters that arose from NMF clustering and not by the individual factors themselves. Initial subtyping approaches have explored the use of immunohistochemistry (IHC) for these factors ^9,10,40^, although this approach is limited by the tissue requirements, challenges in quantitation, heterogeneity in staining, and the observation that no single marker specifically can unequivocally define each subgroup ^9,40^. Additionally, YAP1 was initially proposed to define a distinct subtype itself ^8^ but on further analysis was found to be absent or expressed only at low levels in tumors (typically in the stroma or in the NSCLC component of mixed tumors), although a subpopulation of YAP1 positive cells may emerge in the setting of resistance^9,10,20,41^. Intriguing results from the SWOG1929 trial, a phase II trial assessing the addition of the PARP inhibitor talazoparib to atezolizumab maintenance in ES-SCLC highlighted that biomarker-driven trials in SCLC are possible, even with stratification based on limited tissue, as this trial required SLFN11 positive IHC for enrollment ^42^. Therefore, IHC remains to be an important method for biomarker assessment in SCLC but also for understanding of heterogeneity. Consequently, tissue-based biomarker assessment can guide clinical treatment decisions and the use of an mRNA-based approach can be implemented for SCLC subtyping. However, technical challenges and tissue limitations persist, and thus classification is not possible for all samples as is the analysis of longitudinal samples.

Consequently, we and others hypothesized that DNA methylation might overcome these limitations by providing a more robust classification method as well as enabling a liquid biopsy option. Indeed, DNA methylation has been reported to distinguish ASCL1 and NEUROD1 driven tumors as well as subtypes independent of those transcription factors ^21,43^ and to be associated with drug response ^44^. In addition, DNA methylation has also been implicated in phenotypic regulations like EMT ^45,46^. DNA methylation is highly dysregulated in cancer with transcription factors being particularly regulated by DNA methylation ^47^, making it highly relevant in transcriptionally-defined cancer subtypes like in SCLC.

Hence, using a large cohort of clinical SCLC specimen with genome-wide DNA methylation data, we were able to establish a robust classifier to define SCLC subtypes with comparable clinical outcomes to our RNA-based classification. Importantly, the SCLC-DMC was able to classify tumor samples that failed classification using RNA suggesting potential advantages of DNA methylation over gene expression signatures. Even more, the preservation of DNA methylation patterns in cfDNA is of particular interest as it allows the classification from liquid biopsies. Indeed, our data show limited differences between cfDNA methylation and DNA methylation in the primary tumor. This is critical in SCLC where tumor tissue is limited but high amounts of cfDNA can be isolated ^48^. Thus, the use of cfDNA to identify disease subtypes could rapidly facilitate clinical implementation. DNA methylation has previously been used for detection of SCLC, as well as for its differentiation to other cancers from liquid biopsies ^49^, findings we replicated here by utilizing a commercial DNA methylation assay that incorporates limited DNA methylation sites ^24^.Future assays might be able to combine both, the detection of SCLC for initial diagnosis with the subtyping, to enable a liquid-first rapid therapy initiation, which is especially important in rapidly progressing SCLC ^50^. Additionally, DNA methylation is increasingly used to detect tumor DNA in plasma which could serve as predictor of response to therapy. Consequently, longitudinal plasma samples are critical to track tumor evolution during treatment and serve as early markers of treatment response and relapse ^18,51^.

Furthermore, our study provides new insights into the epigenetic regulation of SCLC subtypes. Interestingly, SCLC-P was consistently hypomethylated in our both primary tissue cohorts, while the other subtypes demonstrated more variability between the two clinical cohorts that will require further investigation. However, our analysis only allowed to investigate DNA methylation and gene expression differences while many epigenetic processes contribute to different SCLC phenotypes ^52^. Importantly, we highlighted strong differences between primary tumor samples and cell lines as well as differences in expression of epigenetic enzymes that might contribute to those differences. Strikingly, SCLC-P cell line models exhibited hypermethylated phenotypes compared to primary tumors. Intriguingly, in this shift in global methylation was coincident with significantly increased expression of the SUV39H1-HP1-DNMT3A/B axis, along with several other methyltransferases, not seen in SCLC-P tissue samples. It is possible that tumor extrinsic factors, such as the tumor microenvironment, play key roles in shaping global methylation patterns in SCLC as has been reported in other cancers^53^. Thus, in cell only systems, such as in vitro cell culture, absence of these factors produces global shifts in tumor methylation patterns. Changes in gene expression and global DNA methylation have also been reported during tumor sphere formation *in vitro* as well as compared to primary tumor, suggesting that cell line cultivation in SCLC might impact gene expression and epigenetic regulation ^54–56^. Considering that cell lines are often used as model for further investigation, it will be important to clarify how representative cell lines are in SCLC to allow robust *in vitro* studies.

Previous work based on mouse models already demonstrated that SCLC subtypes may shift, and that tumors may evolve towards greater heterogeneity, under the selection pressure of different treatments. ^20^ In this study, we confirm the heterogeneity of SCLC subtypes during treatment, as we observed a switch to an inflamed subtype in a large proportion of ASCL1^+^ samples at progression. This finding was supported by the notion that the switch to a more inflammatory phenotype was accompanied by profound changes in the promoter methylation of genes controlling immune cell recruitment, interferon responsiveness and production, as well as inflammatory gene transcription. These findings support the ability of frontline etoposide, platinum, and immunotherapy (EP+IO) therapies to “reawaken” tumor-immune crosstalk in a subset of tumors, including those not initially “inflamed” or SCLC-I. In line with this, we also observed that some samples with SCLC-A or SCLC-N have inflammatory features suggesting that inflammatory states also exist in those samples, in line with recently presented data ^57^. Understanding how some tumors evolve to a more “inflamed” state but still progress clinically will be essential for identifying treatment regimens that can successfully harness the immune system for increased tumor control. Additionally, it is critical to further establish longitudinal collection of SCLC specimens to enable better understanding of evolution and gain deeper insights into SCLC subtype plasticity.

The capability of our system to identify those changes further highlights the power of for liquid-biopsy guided surveillance during cancer treatment in SCLC. Furthermore, it is likely that a cfDNA-specific classifier could be further refined to take into account cfDNA-specific attributes (e.g. background cfDNA methylation) which could further enhance its accuracy.^58^ Likewise, confirmation of our findings in additional independent clinical cohorts is critical for clinical implementation, and will also further clarify reliability in the rare SCLC-P and SCLC-I subtypes. Additional analysis will also need to take into account limitations in ctDNA fraction and will need to establish clear analytical parameters to allow a precise classification of SCLC subtypes in a clinical setting. Likewise, the analysis of gene expression changes from liquid biopsy specimen have not been limited to DNA methylation analysis but also other approaches, assessing the distribution of cfDNA fragments across the genome, like the DELFI ^59^ or the EPICseq method ^23^ to enable fragmentomics-based analysis of SCLC. In addition, the development of highly sensitive nucleosome-capture methods ^22^ have also demonstrated high performance for SCLC subtyping and detection. Future assays might consequently deviate from DNA methylation approaches or might incorporate a combination of different approaches for improved performance ^60^.

Taken together, our approaches using gene expression data as well as DNA methylation in SCLC highlight that reliable subtyping in transcriptionally-defined cancer is feasible from tumor specimen as well as by using a methylation-based liquid biopsy assay. Our findings indicate that DNA methylation-based biomarkers using tumor or blood samples can be implemented for the identification of clinically relevant SCLC subtypes, a critical step towards bringing precision, biomarker-directed therapy into the clinic for SCLC and potentially other tumor types.

### Limitations of the study

In this study we do not assess parameters critical for routine implementation of the developed methods such as RNA/DNA quality, minimal tumor content for tissue-based assays as well as the influence of ctDNA content on subtyping performance and minimal ctDNA content for subtyping. Additionally, while we report on LOD and LOB for the epicheck assay designed to detect SCLC from previously undiagnosed individuals, we do not assess LOD and LOB for our 7-methylation site assay designed to assess ctDNA fraction. Consequently, additional studies are required to translate this method into a validated assay with strict analysis criteria. Furthermore, the limited amount of SCLC plasma samples with matched tissue required to assess performance required us to rely on cross-validation instead of validating the results in independent cohorts. Gathering additional cohorts from various resources and regions is critical to assess the robustness of the methods (for both tissue and plasma) and will be subject of future studies. Lastly, while we observed differences in drug response in cell line models according to SCLC subtypes, final validation of clinical validity of SCLC subtyping is pending prospective clinical trials.

[The Key Resources Table could be either included here OR uploaded as a separate file] Key Resources Table

### Key Resources Table

**Table:** 

REAGENT or RESOURCE	SOURCE	IDENTIFIER
Biological samples		
Human FFPE specimen	This paper	N/A
Human blood plasma specimen	This paper	N/A
Critical commercial assays		
SMARTer Seq V3	Takara	#634487
RNA TruSeq RNA Exome	Illumina	20020189
Ovation RRBS Methyl-Seq	Tecan	0553-32
MagMAX™ FFPE DNA/RNA Ultra Kit	Applied Biosystems	A31881
Apostle MiniMax High Efficiency Cell-Free DNA Isolation Kit	Apostle Bio	A17622-250
Chemicals, peptides, and recombinant proteins		
Cisplatin	MD Anderson Pharmacy	N/A
Software and algorithms		
R v4.2.1	R foundation for statistical computing	https://www.r-project.org/
Caret	Max Kuhn	https://topepo.github.io/caret/
xGBoost	Chen et al. ^61^	https://github.com/dmlc/xgboost/tree/36ad160501251336bfe69b602acc37ab3ec32d69
Trimmomatic	Bolger et al. ^62^	https://github.com/usadellab/Trimmomatic
Bismark	Felix Krueger	https://github.com/FelixKrueger/Bismark
Salmon	Patro et al.^63^	https://github.com/COMBINE-lab/salmon
CAMDAC	Cadieux et al. ^28^	https://github.com/VanLoo-lab/CAMDAC
Deposited data		
RNA-seq of SCLC specimen	This paper	phs003416.v1.p1
RRBS of SCLC specimen	This paper	phs003416.v1.p1
ULP-WGS of SCLC plasma specimen	This paper	phs003416.v1.p1
RRBS of plasma specimen	This paper	phs003416.v1.p1
RRBS of cell line specimen	This paper	GSE241673
RNA-seq of SCLC Specimen	George et al. ^25^	EGAS00001000925
RNA-seq of SCLC Specimen	IMPower133 ^10^	EGAS00001004888
RNA-seq of cell lines	NCI Cell Miner ^29^	https://discover.nci.nih.gov/rsconnect/SclcCellMinerCDB/
RNA-seq of cell lines	GDSC ^30^	https://www.cancerrxgene.org/downloads/bulk_download
Experimental models: Cell lines		
Human cell line: H1694	ATCC	Cat # CRL-5888
Human cell line: H446	ATCC	Cat # HTB-171
Human cell line: H2171	ATCC	Cat # CRL-5929
Human cell line: H847	ATCC	Cat # CRL-5846
Human cell line: H82	ATCC	Cat # HTB-175
Human cell line: NJH29	Kindly provided by Dr. Julien Sage (Stanford University, Stanford, CA)	N/A
Human cell line: H524	ATCC	Cat # CRL-5831
Human cell line: DMS273	Sigma Aldrich	Cat # 95062830-1VL
Human cell line: SHP-77	ATCC	Cat # CRL-2195
Human cell line: H865	ATCC	Cat # CRL-5849
Human cell line: H2330	ATCC	Cat # CRL-5940
Human cell line: H1522	ATCC	Cat # CRL-5874_FL
Human cell line: H2196	ATCC	Cat # CRL-5932
Human cell line: DMS53	ATCC	Cat # CRL-2062
Human cell line: H146	ATCC	Cat # HTB-173
Human cell line: DMS79	ATCC	Cat # CRL-2049
Human cell line: H1876	ATCC	Cat # CRL-5902
Human cell line: H209	ATCC	Cat # HTB-172
Human cell line: H2108	ATCC	Cat # CRL-5984_FL
Human cell line: H378	ATCC	Cat # CRL-5808
Human cell line: H1688	ATCC	Cat # CCL-257
Human cell line: H2195	ATCC	Cat # CRL-5931
Human cell line: H1436	ATCC	Cat # CRL-5871
Human cell line: H345	ATCC	Cat # CRL-5846
Human cell line: H2198	ATCC	Cat # HTB-180
Human cell line: H735	ATCC	Cat # CRL-5978
Human cell line: H69	ATCC	Cat # HTB-119
Human cell line: H250	ATCC	Cat # CRL-5828
Human cell line: H1963	ATCC	Cat # CRL-5982
Human cell line: H187	ATCC	Cat # CRL-5804
Human cell line: H1105	ATCC	Cat # CRL-5856
Human cell line: H128	ATCC	Cat # HTB-120
Human cell line: H510A	ATCC	Cat # HTB-184
Human cell line: H1672	ATCC	Cat # CRL-5886
Human cell line: DMS153	ATCC	Cat # CRL-2064
Human cell line: H1417	ATCC	Cat # CRL-5869
Human cell line: H748	ATCC	Cat # CRL-5841
Human cell line: H2029	ATCC	Cat # CRL-5913
Human cell line: H1238	ATCC	Cat # CRL-5859
Human cell line: H740	ATCC	Cat # CRL-5840
Human cell line: H774	ATCC	Cat # CRL-5842
Human cell line: H2081	ATCC	Cat # CRL-5920
Human cell line: H2141	ATCC	Cat # CRL-5927
Human cell line: H2107	ATCC	Cat # CRL-5983_FL
Human cell line: CORL88	Sigma Aldrich	Cat # 92031917-1VL
Human cell line: H889	ATCC	Cat # CRL-5817
Human cell line: H1092	ATCC	Cat # CRL-5855
Human cell line: H719	ATCC	Cat # CRL-5837
Human cell line: H1836	ATCC	Cat # CRL-5898
Human cell line: H1618	ATCC	Cat # CRL-5879
Human cell line: H526	ATCC	Cat # CRL-5811
Human cell line: H211	ATCC	Cat # CRL-5824
Human cell line: H196	ATCC	Cat # CRL-5823
Human cell line: H841	ATCC	Cat # CRL-5845
Human cell line: DMS114	ATCC	Cat # CRL-2066
Human cell line: H1930	ATCC	Cat # CRL-5906
Human cell line: H1048	ATCC	Cat # CRL-5853
Human cell line: H1341	ATCC	Cat # CRL-5864
Human cell line: H2227	ATCC	Cat # CRL-5934

## STAR Methods

### Resource Availability

#### Lead Contact

Further information and requests for resources and reagents should be directed to and will be fulfilled by the lead contact, John V. Heymach (jheymach@mdanderson.org).

#### Materials Availability

This study did not generate new unique reagents. Cell lines used in this manuscript have been retrieved and are available from ATCC.

### Experimental Model and Study Participant Details

#### Patient Selection

Patients in this study were included in two cohorts. In cohort 1, 105 patients have been selected after pathological examination of the tissue quality. All patients in this cohort were consented to the GEMINI protocol at the UT MD Anderson Cancer Center (UT MDACC). In cohort 2, 74 patients were included from the UT MD Anderson Cancer Center, the Hospital del Mar, Barcelona, Spain, Vanderbilt Medical Center, Nashville, TN USA, and LPCE Biobank Cote d’Azur (BB-0033-00025), Nice, France. For 15/74 patients in cohort 2, plasma and previously extracted RNA was included. For those patients, only RNA-seq and plasma DNA methylation was performed but no tissue DNA methylation due to the absence of tissue for DNA extraction. All patients provided written informed consent. Each sample was required to have > 100 tumor cell in each specimen, and at least 2 slides of tissue sections was required for inclusion in the study. All patients provided written informed consent prior to study enrollment and the study complied with the declaration of Helsinki.

##### Patient samples

Formalin-fixed and paraffin embedded (FFPE) specimen were used from all patients. At least two sections of 5-8µm were used. Each slide were analyzed by a board certified pathologist to contain at least 100 tumor cells. Blood was obtained by phlebotomy and plasma was processed within 6h of blood draw. 1-2ml of plasma were used for each patient.

#### Clinical Data

Clinical data was retrieved from the GEMINI database which includes clinical data obtained during treatment at the UT MDACC and consent was provided for accessing the clinical data. Additional data was retrieved manually and reviewed by three board-certified oncologists. For the analysis of survival, overall survival was calculated by time from date of diagnosis to death and patients with lost follow-up were censored at the date where the last information was obtained. Survival analysis was performed using Kaplan-Meier analysis and cox-proportional hazard ratio estimation using the survminer package ^64^ in R ^65^.

#### Cell line Samples

The human SCLC cell lines H1694, H446, H2171, H847, H82, NJH29, H524, DMS273, SHP-77, H865, H2330, H1522, H2196, DMS53, H146, DMS79, H1876, H209, H2108, H378, H1688, H2195, H1436, H345, H2198, H735, H69, H250, H1963, H187, H1105, H128, H510A, H1672, DMS153, H1417, H748, H2029, H1238, H740, H774, H2081, H2141, H2107, CORL88, H889, H1092, H719, H1836, H1618, H526, H211, H196, H841, DMS114, H1930, H1048, H1341, H2227 were obtained from ATCC (Manassas, VA) or Sigma Aldrich (St. Louis, MO). The patient-derived xenograft cell line NJH29 was kindly provided by Dr. Julien Sage (Stanford University, Stanford, CA). Cells were grown in suggested media supplemented with 5% fetal bovine serum and 1% penicillin/streptomycin and maintained in a 37°C humidified chamber with 5% CO2. Cells were passaged less than six months from the time they were received, regularly tested for Mycoplasma contamination and routinely subjected to DNA fingerprinting.

For the treatment with chemotherapy, H1876 and H2195 cells were cultivated in HITES with 5% fetal bovine serum and 1% penicillin/streptomycin. They were treated with 2µM cisplatin for 0, 2, 5, 9 days, respectively.

### Method Details

#### SCLC detection using cfDNA

Detection of SCLC has been performed using a commercial PCR based assay. Initial validation has been performed previously ^24^. Sample inclusion, assay execution and data analysis has been performed as highlighted previously. However, 288 additional specimens have been included in this analysis. Furthermore, new cut-offs specifically for the detection of SCLC have been selected in this study. Level of detection (LOD) and level of blank (LOB) was determined by 22 replicates of an unmethylated plasmid DNA that contain the cloned markers spiked into healthy human cfDNA in order to establish the limit of blank (LOB) for each marker separately (the average LOB across the markers was 1:249,281). Totally 35ng of DNA was used for the spike-in experiment of which 3.5ng of DNA was used per qPCR reaction for each of the six markers. For the assessment of LOD, we spiked the unmethylated DNA together with DNA that is methylated in these 6 markers from a human lung cancer cell line into 35ng of the healthy human plasma cfDNA at a dilution of 1:10,000 (methylated:unmethylated). All 22 replicates detected DNA methylation in the six markers demonstrating a LOD of at least 1:10,000.

#### Nucleic Acid Extraction

For the nucleic acid extraction, at least two slides of FFPE tissue samples were cut at 5-8µm each. For each sample, tumor area was highlighted by a board-certified Pathologist and macrodissection was used prior to extraction in cohort 1 but not cohort 2, if necessary. For combined RNA and DNA extraction, the MagMAX FFPE DNA/RNA Ultra Kit (Thermo Fisher Scientific, A31881) was used following the manufacturer’s protocol. DNA concentration was assessed using the Qubit 1X dsDNA HS Assay Kit and a Qubit 2.0 fluorimeter. For RNA, concentration was measured using the Qubit RNA high sensitivity (HS) assay kit. RNA quality was analyzed using the Agilent RNA 6000 Pico kit on a 2100 Bioanalyzer.

For cfDNA extraction, 1-3 ml Plasma obtained in Streck Cell-Free DNA BCT tubes was used for each sample. Plasma was obtained within 6h of phlebotomy by spinning the blood for 10 minutes at 1800xg followed by a second centrifugation step of the isolated plasma for 10 minutes at 2000xg. Both centrifugation steps were performed in swing-bucket rotors. cfDNA was extracted using the Apostle MiniMax High Efficiency Cell-Free DNA Isolation Kit (Apostle Inc). cfDNA concentration was assessed using the Qubit 1X dsDNA HS Assay Kit and a Qubit 2.0 fluorimeter.

#### RNA-seq

For cohort 1, 85 samples have been selected for RNA sequencing. All samples were treated with DNase treatment using DNase I (ThermoFisher, Massachusetts, USA) prior to RNA-seq to reduce DNA contamination that might interfere with downstream results. Library generation using the SMARTer Stranded Total RNA-seq Kit V3 (Takara Bio USA Inc., California, USA) was performed following the manufacturer’s instructions. Final library quantity was measured by KAPA SYBR FAST qPCR and library quality was evaluated using a TapeStation D1000 ScreenTape (Agilent Technologies, CA, USA). Libraries were sequenced on an Illumina NovaSeq instrument (Illumina, California, USA) with a read length configuration of 150 PE for 80M PE reads per sample (40M clusters). Fastq files were quality trimmed using trimmomatic and aligned to the GRCh38 transcriptome using salmon v1.6.0.

For cohort 2, 57 samples have been submitted for RNA-seq using the Illumina RNA Access hybrid capture-based protocol. All samples were treated with DNAse I prior to library generation according to manufacturer’s protocol. Sequencing was performed on an Illumina NovaSeq instrument with 100M PE configuration. 40M reads were used for each sample. Fastq files were quality trimmed using trimmomatic and aligned to the GRCh38 transcriptome using salmon v1.6.0.

#### RRBS

To analyze DNA Methylation across the genome, RRBS (Reduced Representation Bisulfite Sequencing) ^66,67^ was utilized using the Ovation RRBS Methyl-Seq kit (Tecan Group Ltd., Zurich, Switzerland). To account for the highly degraded DNA from FFPE and plasma samples, the material was first treated with one unit of Shrimp Alkaline Phosphatase (New England Biolabs, Ipswich, MA) to remove phosphorylated DNA which might interfere with downstream analysis ^34^. Briefly, 0.1 – 100ng of genomic DNA was digested using MspI, and Illumina-compatible cytosine-methylated adaptor were ligated to the enzyme-digested DNA. For lower concentrations of DNA, adapters were diluted 1:40 to 1:120, in order to decrease the representation of randomly fragmented DNA and adapter-dimers in the final library. RRBS libraries were then visualized using Bioanalyzer High Sensitivity DNA chips (Agilent, Santa Clara, CA), and those passing QC were subsequently sequenced as 100bp paired-end reads on an Illumina NovaSeq instrument with a target sequencing depth of 300M PE reads (150M clusters). After sequencing, Fastq files were obtained and adapters were trimmed using trimmomatic. Alignment and retrieval of DNA Methylation (in percent of total methylated Cytosines) was performed using Bismark v 0.22 ^68^ against the GRCh38 human genome. Samples with < 50% mapping rate and, < 60M aligned reads were excluded from further analysis. Finally, cytosines with coverage < 10 were filtered out to assure high confidence DNA Methylation analysis.

For cell lines, 100ng of RNA was used using the Ovation RRBS Methyl-Seq kit (Tecan Group Ltd., Zurich, Switzerland) as for the clinical samples but without the initial phosphatase step. Sequencing was performed in a single Read 57 bp configuration on a Illumina HiSeq 3000 sequencer. Data processing was performed likewise using Bismark v 0.22. Annotations of methylated regions was performed using the annotatr ^69^package and the Hg38 database.

Deconvolution of tumor intrinsic signals in cohort 2 was performed using the Copy number-aware deconvolution of tumor-normal DNA methylation (CAMDAC) algorithm as published previously ^28^.

#### ULP-WGS

Library preparation was performed using the KAPA HyperPrep Kit with Library Amplification product KK8504) and IDT’s duplex UMI adapters (KAPA Biosciences). Sequencing is performed on a NovaSeq 6000 with 2x 150bp configuration and a target sequencing depth of ~ 0.3x.

In order to define DNA methylation sites that are associated with general ctDNA content, we correlated DNA methylation sites against their reported cDNA content using ULP-WGS. Only sites with R^2^ > 0.65, slope between 0.9 and 1.1 and intercept between -10 and 10 were selected. After manual analysis, seven sites have been selected: “chr12:27974490”, “chr1:7236563”, “chr17:29139387”, “chr19:128737209”, “chr2:10401557”, “chr21:34669078”, “chr21:45590104”. ctDNA content was calculated by averaging the methylation level across all seven sites for each sample.

#### Generation of Predictive Models for Classification using RNA-seq

We hypothesized that using gene ratios of one gene over another gene might be more robust to classify SCLC across different datasets than using the single expression value. For this purpose, we combined the data retrieved from George et al. comprising of surgical SCLC specimen and the data from the IMPower133 clinical trial as published in Gay CM et al ^10^. While for the latter only limited genes were published, we filtered for genes that were present in both datasets that served as training set. We used ROC analysis to define the genes which were mostly associated with one of the four subtypes by analysing the association of each respective gene with each of the four subtypes. For each of the four subtypes, the Top 50 genes with the highest area under the curve in ROC analysis have been selected for model generation. Due to some overlaps across the genes selected, finally 181 genes were used (Table S1). We then created all different gene ratios of those genes. To select highly relevant gene ratios, we created predictive models, incorporating randomly selected 20 gene ratios per model with 500 distinct models for each of the four subtypes (totally 2000 models created). For the training, the caret package ^70^ in R was used, and extreme gradient boosting with DART (Dropout Additive Regression Trees) ^71^ was utilized with repeated cross validation with a 5-fold split and 20 repeats during training. Those models were then used to define the subtypes in our clinical dataset. In order to obtain the most generalized subtype classification, we used all models for the prediction and if >50% of the models agreed on the subtype, the subtype was called based on this consensus classification. Samples with less than 50% agreement are called “equivocal” as a clear classification could not be obtained with our current methodology. Consensus as well as subtyping for each sample is provided in Table S1.

#### Generation of Predictive Models for Classification using DNA Methylation Data

To generate models with broader applicability, we combined data from the cell lines and our clinical GEMINI cohort in order to tune models to work across different sample types. The selected DNA Methylation sites were filtered to be present in both datasets. Furthermore, only methylation sites with <= 10% missing data were used. Following, we performed ROC analysis on the combined set to select the methylation sites that had the highest association with one of the four subtypes by analysing the association of each DNA methylation site with each of the four subtypes. We selected based on the following criteria; For SCLC-A: AUC >= 0.7 & difference to other subtypes >= abs(25%) (N = 199), for SCLC-N: AUC >= 0.7 & diff >= abs(30) (N = 127), for SCLC-P: AUC >= 0.8 & diff >= abs(35) (N = 194), for SCLC-I: AUC >= 0.7 & diff >= abs(30) (N = 293; Table S3). Initially, we analyzed the influence of number of methylation sites and performance by selecting, 1, 2, 3, 4, 5, 10, 20, 30, 40, 50, 100 methylation sites randomly training 100 models for each of the combinations. We analyzed the accuracy for each of those models on the training and the testing set (Figure S6A). Based on this analysis, for each of the four subtypes we created models by randomly selecting 10, 50, or 100 methylation sites per subtype per model for our final classifier. For each number of methylation sites, 500 models were created using xgboost with DART leave-one-out cross validation (LOOCV) using the training set. Similar to the RNA-seq approach, a subtype was called when >50% agreed on the subtype. If < 50% agreement was achieved, the subtype was classified as “equivocal” due to the lack of consensus. The classification for each sample is provided in Table S3.

For models predicting subtypes using cfDNA, the same methylation sites were used but filtered for presence in the cfDNA dataset. Similarily to tissue models, we used xgBOOST with DART and LOOCV and trained 500 models per subtype. The consensus approach was applied. The classification for each sample is provided in Table S3.

### Quantification and Statistical Analyses

All analysis have been performed in R v4.1.1 ^65^. Binning of the genome was performed based on the BSgenome.Hsapiens.NCBI.GRCh38 database ^72^ using a tile width of 100bp or 100,000 bp cutting the last tile of each chromosome. DNA methylation across each tile was averaged excluding missing data. To analyse the genome-wide methylation per subtype, the mean methylation per tile per sample was further averaged per subtype. The rolling average of 500 bins (= 50Mbp) was calculated using the ‘rollmean’ function in the R zoo package ^73^.

In order to annotate the methylation sites to regions in the genome associated with genes, the annotatr package has been used ^69^. The following regions have been annotated based on the GRCh38 genome: “hg38_genes_promoters”, “hg38_genes_exons”, “hg38_genes_introns”, “hg38_genes_1to5kb”, “hg38_genes_5UTRs”, “hg38_genes_intergenic”, “hg38_genes_3UTRs”, “hg38_genes_firstexons”, “hg38_genes_intronexonboundaries”, “hg38_genes_exonintronboundaries”.

Association of DNA methylation sites or regions has been performed using pROC ^74^. Cut-offs were calculated using Youden’s J and sensitivity and specificity has been calculated based on the pre-calculated cut-off. For the calculations of differences, unless otherwise highlighted, Wilcoxon test has been used with FDR correction for multiple testing using rstatix ^75^.

Figures were created using ggplot2 ^76^ or ComplexHeatmap ^77^. The graphical abstract was created using Biorender.com.

## Supplementary Material

Data S1 RNA-seq data of cohort 1, related to Figure 1.

Data S2 RNA-seq data of cohort 2, related to Figure 2.

Figure S1

Table S1

Table S2

Table S3

## Figures and Tables

**Table 1:** Overview of included patients for the whole cohort, cohort 1 (C1) and cohort 2 (C2)

	Group	All	C1	C2
N		179	105	74
**Age (range)**		66 (26 - 96)	66 (26 - 96)	68 (45 - 82)
**Sex (%)**	F	72 (40%)	57 (54%)	15 (20%)
	M	107 (60%)	48 (46%)	59 (80%)
**RNA-seq [Yes/No]**		142 (79%)	85 (81%)	57 (77%)
**RNA classification (%)** **SCLC-GRC**	SCLC-A	75 (42%)	47 (45%)	28 (38%)
	SCLC-N	25 (14%)	22 (21%)	3 (4%)
	SCLC-P	15 (8%)	4 (4%)	11 (15%)
	SCLC-I	21 (12%)	8 (8%)	13 (18%)
	equivocal	6 (3%)	4 (4%)	2 (3%)
**RRBS [Yes/No]**		124 (69%)	83 (79%)	41 (55%)^¥^
**RRBS classification (%)** **SCLC-DMC**	SCLC-A	78 (44%)	55 (52%)	23 (31%)
	SCLC-N	23 (13%)	20 (19%)	3 (4%)
	SCLC-P	11 (6%)	3 (3%)	8 (11%)
	SCLC-I	11 (6%)	5 (5%)	6 (8%)
	equivocal	1 (1%)	0 (0%)	1 (1%)
**Both [RNA-seq & RRBS]**		100 (56%)	66 (63%)	34 (46%)

¥ For 15/74 samples (21%) only previously extracted RNA was available and thus no RRBS could be performed. Excluding those samples, success rate for RRBS was 72% (41/57) for C2 and 76% (124/164) for the complete data set.

## Data Availability

Code generated in this manuscript can be found at: https://github.com/MD-Anderson-Bioinformatics/SCLC_Subtyping Raw sequencing data generated as part of this manuscript are deposited in dbGap (https://www.ncbi.nlm.nih.gov/gap/) under accession number phs003416.v1.p1. Sequencing data from cell lines are deposited in GEO (https://www.ncbi.nlm.nih.gov/geo/) with accession number GSE241673. Processed RNA-seq data for cohort 1 and cohort are additionally directly provided in this manuscript as data S1 and data S2, respectively. Any additional information required to reanalyze the data reported in this paper is available from the lead contact upon request.
