# Supplementary material for: Tumor- and circulating-free DNA methylation identifies clinically relevant small cell lung cancer subtypes
Source: Cancer Cell. Author manuscript; Available in PMC 2024 Jun 14. (PMC10982990; doi:10.1016/j.ccell.2024.01.001)
Supplement: Figure S1 [file EMS195999-supplement-Figure_S1.pdf]

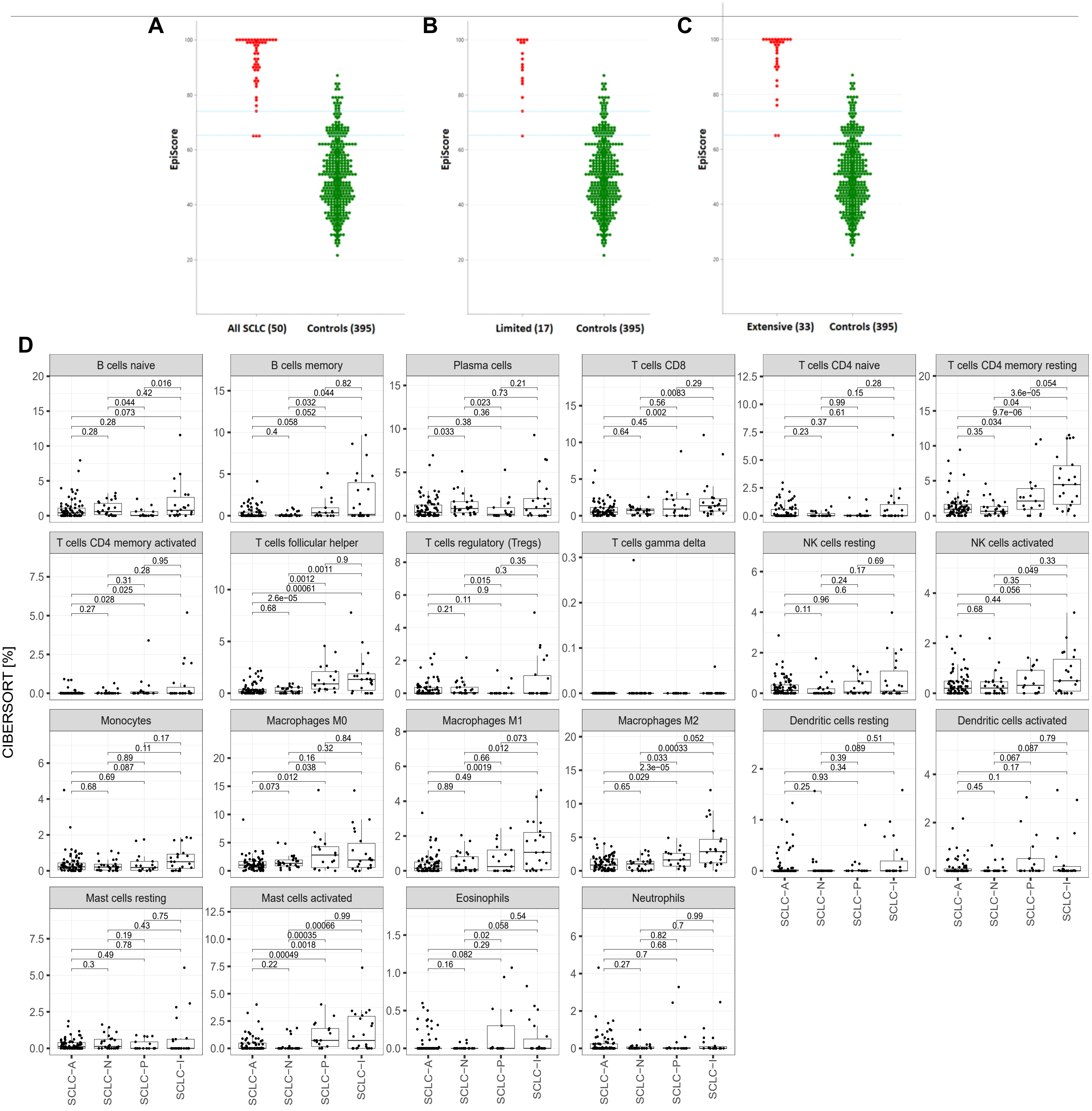

Figure S1: Detection of SCLC in non-cancer control and immune composition of SCLC tumor specimen. Related to Figure 1. Detection of SCLC using the EpiCheck assay for **A** the entire cohort, all cases with SCLC (n=50) and all controls (n=395). **B** Cases with limited stage SCLC (n=17) and all controls (n=395). **C** Cases with Extensive stage SCLC (n=33) and all controls (n=395). the two blue lines represent the two cut-offs of the analysis: Low cut-off at EpiScore 65 and high cut-off at EpiScore 74. **D** CIBERSORT deconvolution of RNAseq data of tumor specimen. Comparison across SCLC subtypes using Wilcoxon test. Boxplot shows the median as thick line, the box highlighting the first and third quartile with the whiskers highlighting 1.5x the interquartile range.

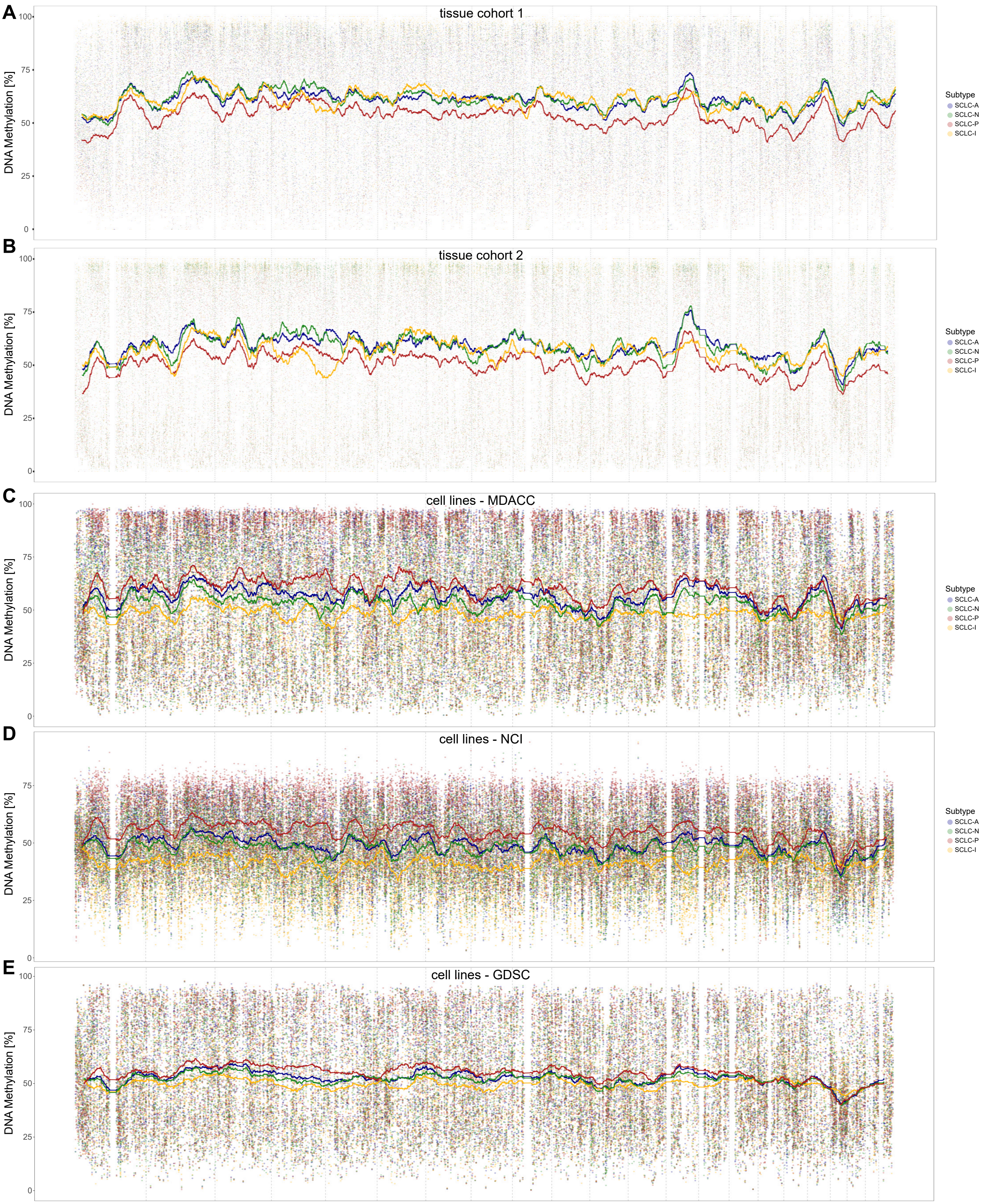

Figure S2: **Subtype-specific DNA methylation in SCLC. Related to Figure 2. A** DNA methylation was assessed using reduced-representation bisulfite sequencing (RRBS) and DNA methylation was averaged per sample and subtype over 100kbp bins (represented by a dot) and the rolling average over 500 bins (= 50mbp) is highlighted in a subset of samples after deconvolution of RRBS data for tumor-intrinsic signals using the CAMDAC method. **B** Analysis of a DNA methylation patterns per subtype in cohort 2. **C** Cell lines RRBS data from the MD Anderson Cancer Center (MDACC). **D** Cell line data from the **I**llumina MethylationEPIC BeadChip from the NCI cell miner project and **E** cell line data from the **I**llumina 450K Methylation Array from the Genomics of Drug Sensitivity in Cancer study (GDSC).

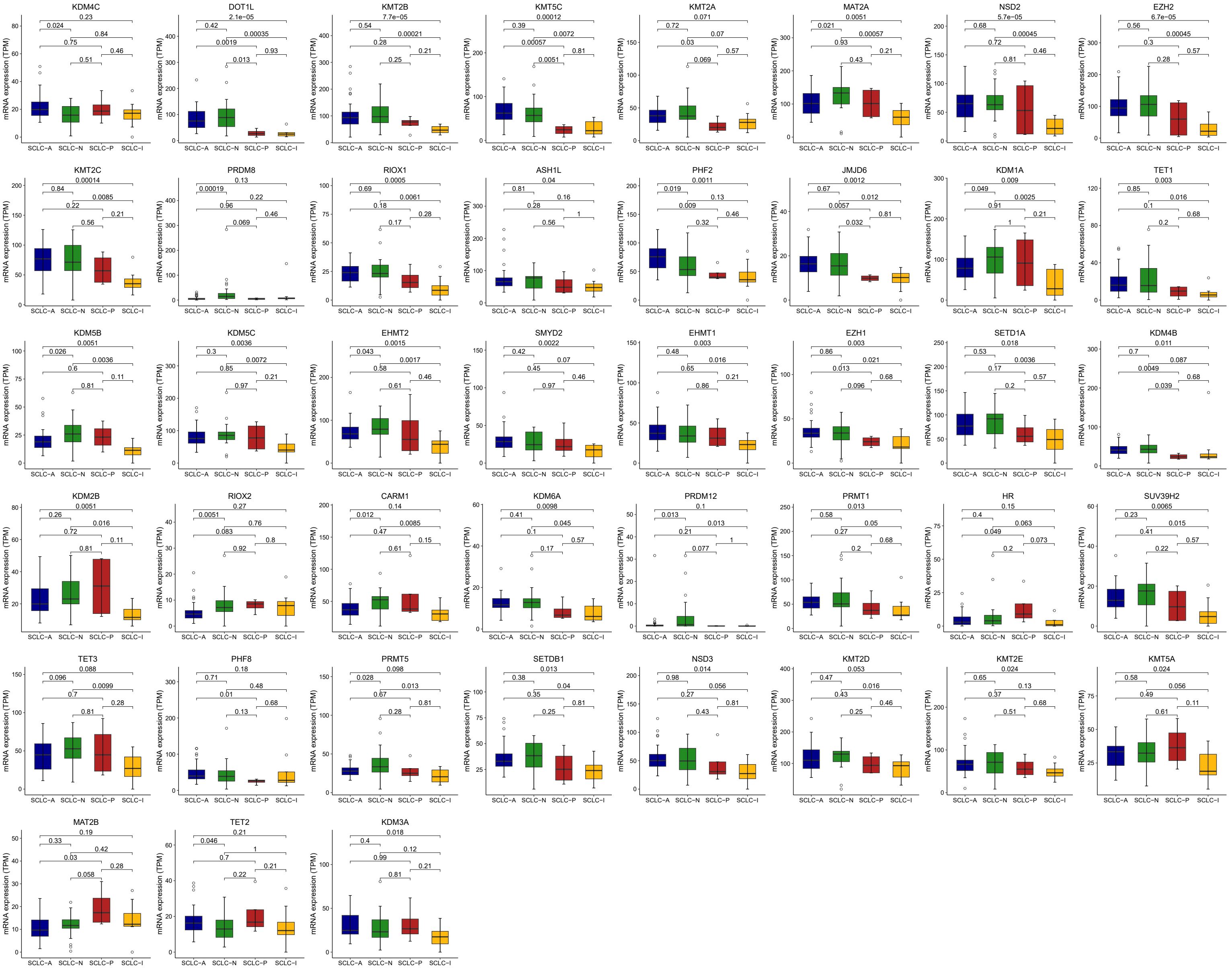

Figure S3: Analysis of differences in gene expression of epigenetic enzymes significantly differentially expressed in SCLC tumor samples of cohort 1. Related to Figure 2. Comparison across SCLC subtypes using Wilcoxon test. Boxplot shows the median as thick line, the box highlighting the first and third quartile with the whiskers highlighting 1.5x the interquartile range.

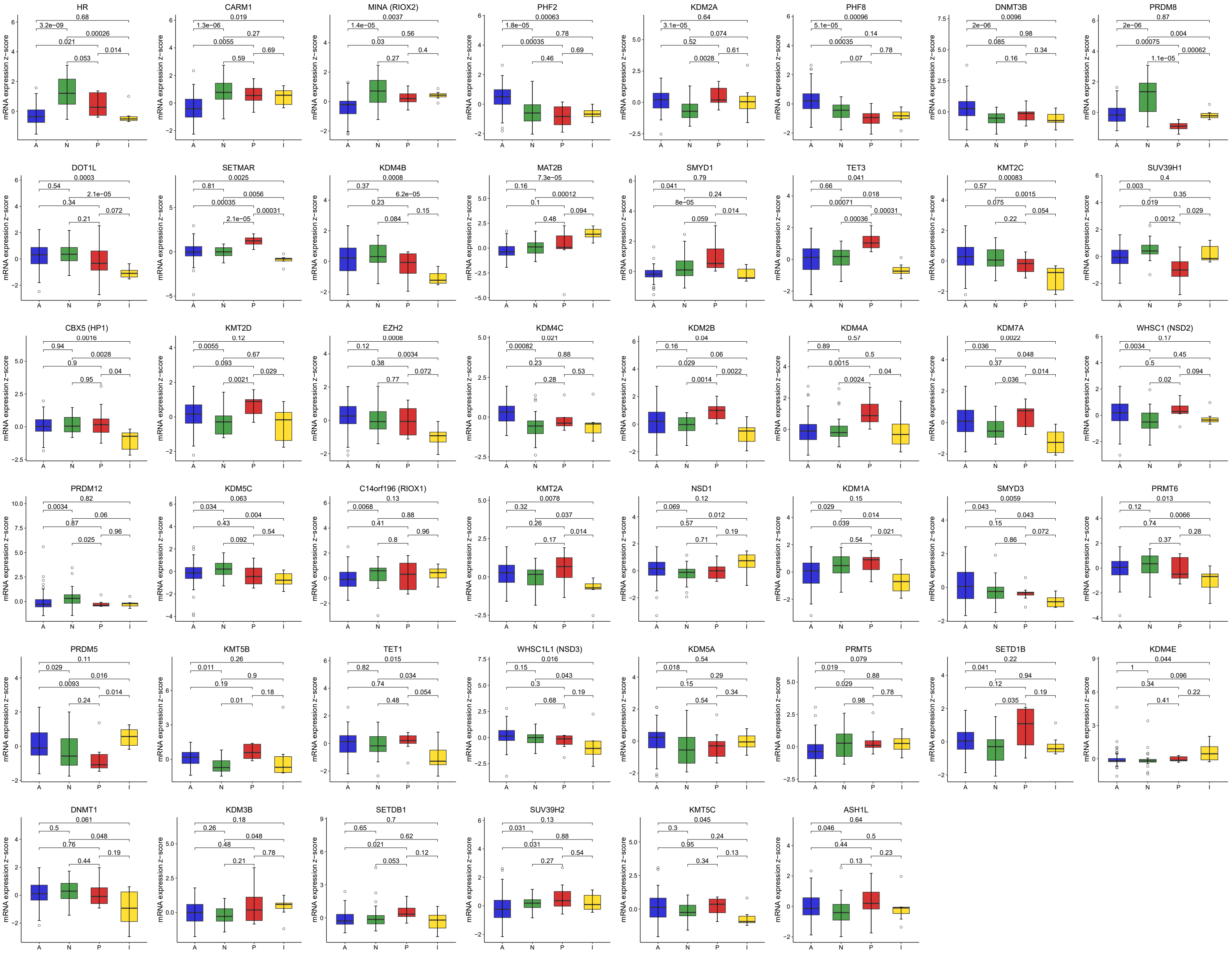

Figure S4: Analysis of differences in gene expression of epigenetic enzymes significantly differentially expressed in SCLC cell lines. Related to Figure 2. Comparison across SCLC subtypes using Wilcoxon test. Boxplot shows the median as thick line, the box highlighting the first and third quartile with the whiskers highlighting 1.5x the interquartile range.

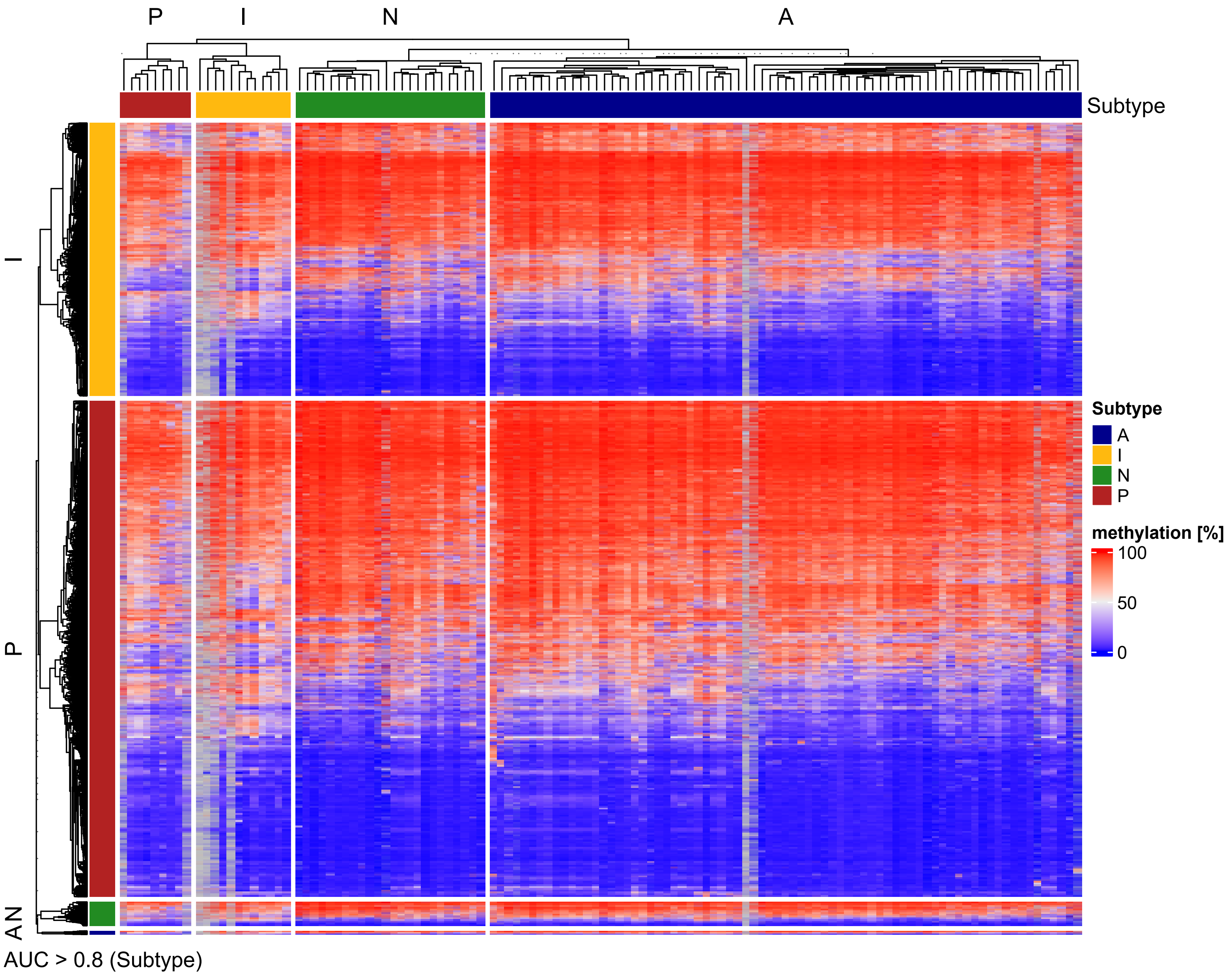

Figure S5: Overview of DNA methylation sites used for tissue classification. Related to Figure 2. Tissue DNA methylation sites with AUROC > 0.8 for each of the four subtypes (each row represents one methylation site separated by subtype with the methylation site is associated. Each column represents one patient separated by SCLC subtype.

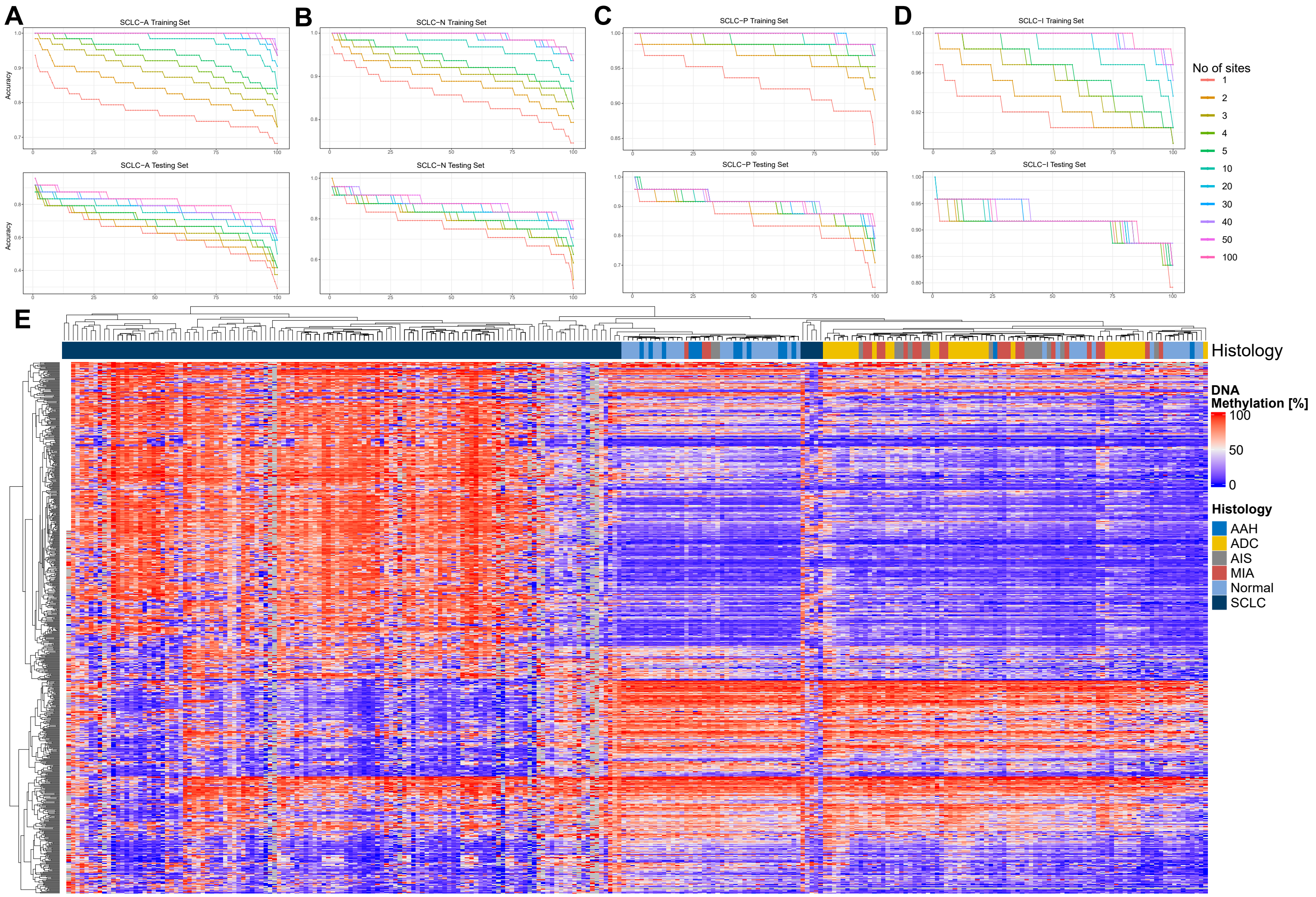

Figure S6: Influence of methylation sites on subtyping and differentiation of lung cancer histologies and non-cancer controls by methylation. Related to Figure 3. Evaluating the influence of number of methylation sites on model accuracy in **A** SCLC-A, **B** SCLC-N, **C** SCLC-P and **D** SCLC-I. For each of the four subtypes, 100 models using 1-100 methylation sites has been trained and accuracy is highlighted for the training and testing set for each of the four subtypes. **E** Analysis of DNA methylation sites used for the SCLC-DMC classifier across SCLC and other lung histologies as well as normal lung tissue. Spearman correlation was used for correlation across samples. AAH: atypical adenomatous hyperplasia; ADC: adenocarcinoma; AIS: adenocarcinoma in situ; MIA: minimally invasive adenocarcinoma.

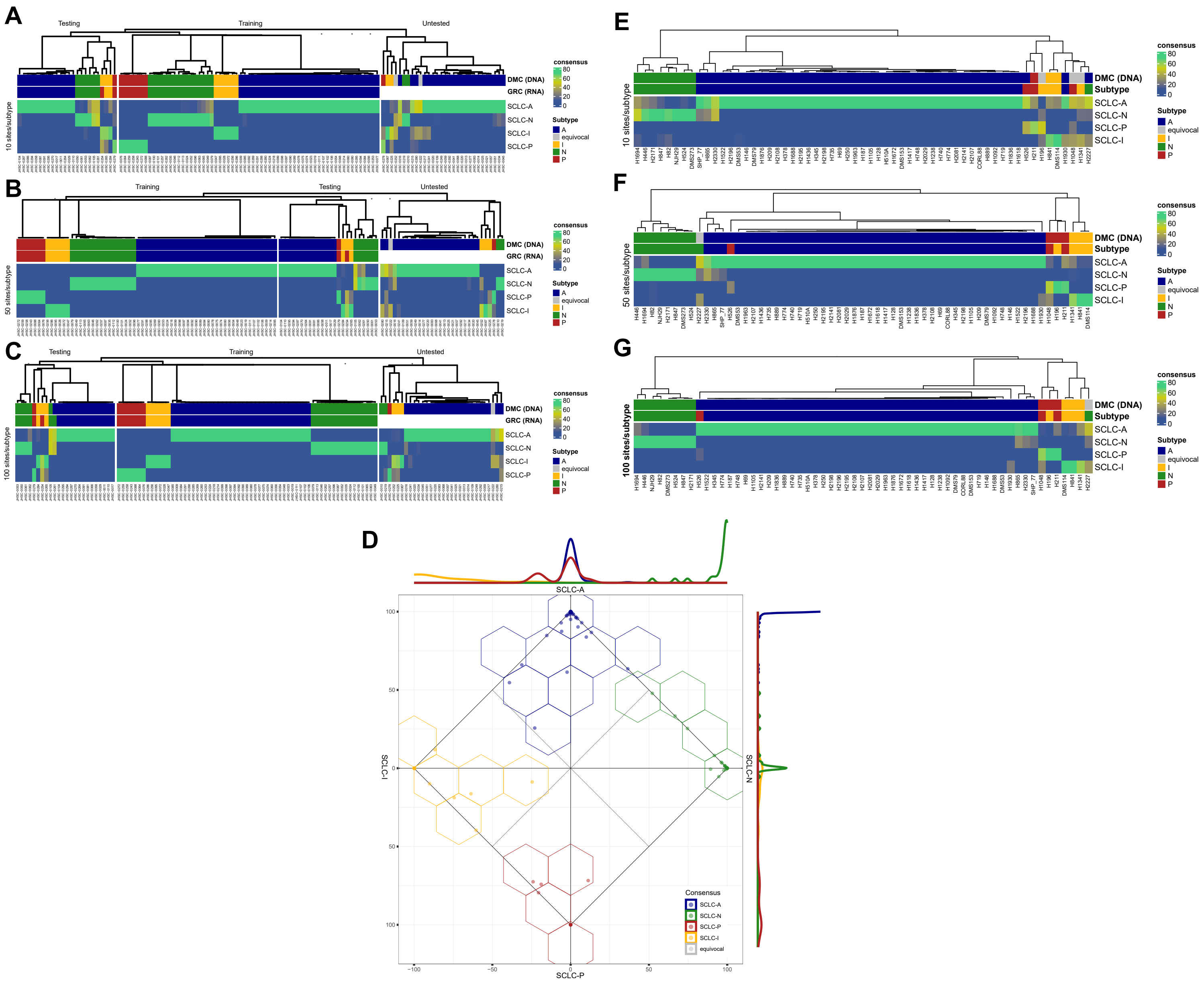

Figure S7: Classification of SCLC specimen from tissue and cell lines. Related to Figure 3. Classification of SCLC tumor specimen using the consensus approach (SCLC-DMC) from DNA methylation using **A** 10 methylation sites per subtype, **B** 50 methylation sites/subtype (note that this figure is similar to main figure 3B but copied for better comparison) and **C** 100 methylation sites per subtype. **D** Characterization of SCLC consensus heterogeneity from classification using the SCLC-DMC approach. The consensus agreement value for each subtype is plotted demonstrating limited overlaps between SCLC subtypes. The line plot at the axis characterizes the distribution of subtypes across the axis based on the DMC consensus approach. All samples analyzed (training, testing and untested set) are included in this analysis. **E-F** Classification of SCLC cell lines using the tumor-specimen trained consensus approach (SCLC-DMC) from DNA methylation using **E** 10 methylation sites per subtype, **F** 50 methylation sites/subtype and **G** 100 methylation sites per subtype.

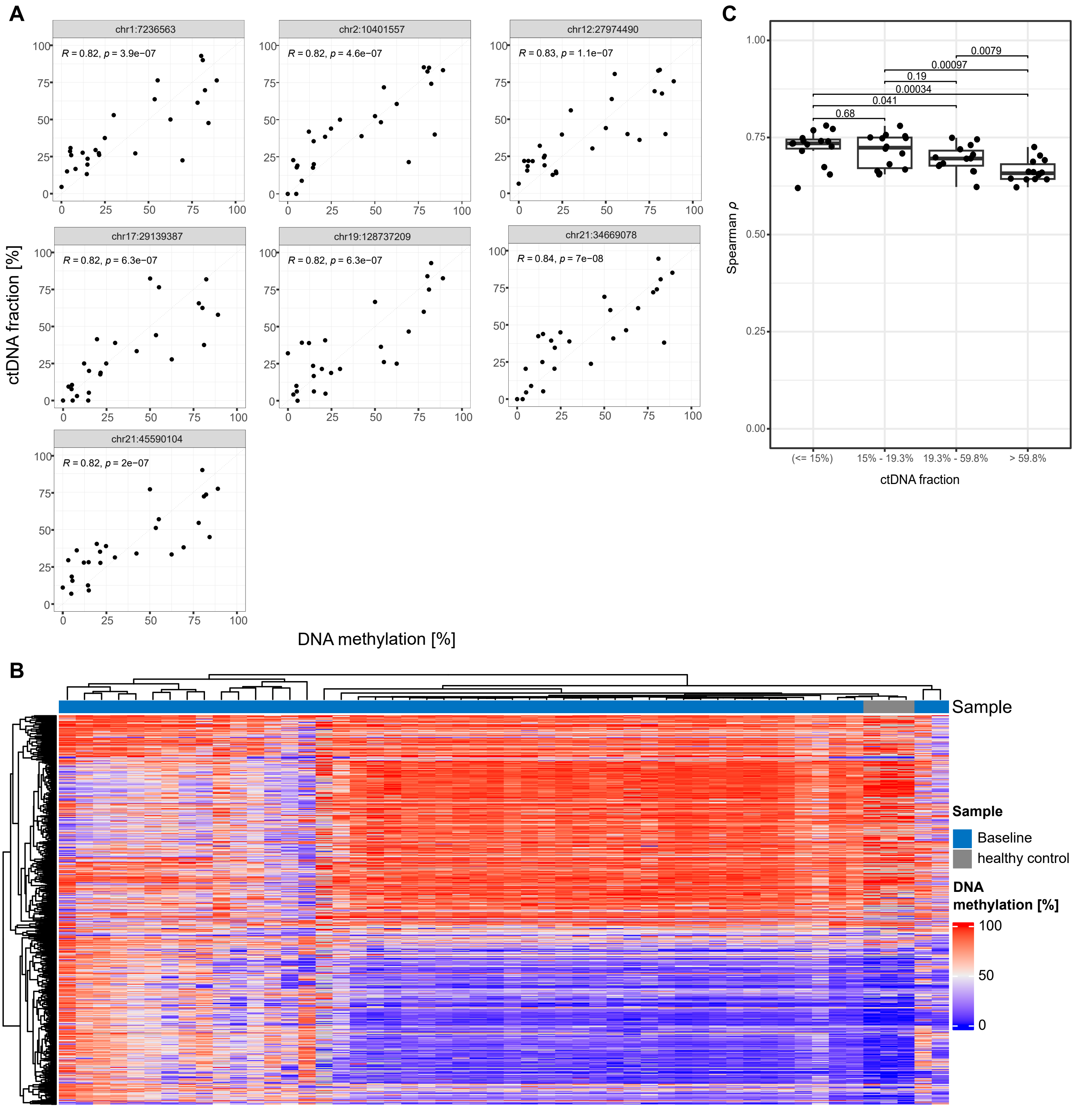

Figure S8: Influence of ctDNA fraction on methylation and comparison to non-cancer controls. Related to Figure 3. A Association of distinct DNA methylation sites with tumor burden as calculated by ULP-WGS. The coefficient of correlation (R) as well as *p*-value of correlation is given. The genomic position based on GRCh38 is highlighted for each methylation site. **B** Analysis of DNA methylation sites used for the SCLC-cfDMC classifier across SCLC as well as health specimen. Spearman correlation was used for correlation across samples. **C** Correlation of SCLC cfDNA methylation compared to mean DNA methylation of no cancer control samples (N = 3) according to ctDNA fraction. Spearman ρ was calculated for each cfDNA baseline sample with known ctDNA content against normal control plasma. Differences are compared against quantiles of ctDNA fraction: first quartile (<= 15% ctDNA fraction), second quantile (15% - 19.3% ctDNA fraction), third quartile (19.3% - 59.8% ctDNA fraction) and fourth quartile (> 59.8% ctDNA fraction). Differences across quartiles are computed using Wilcoxon test and *p*-values are highlighted for each comparison. Boxplot shows the median as thick line, the box highlighting the first and third quartile with the whiskers highlighting 1.5x the interquartile range.

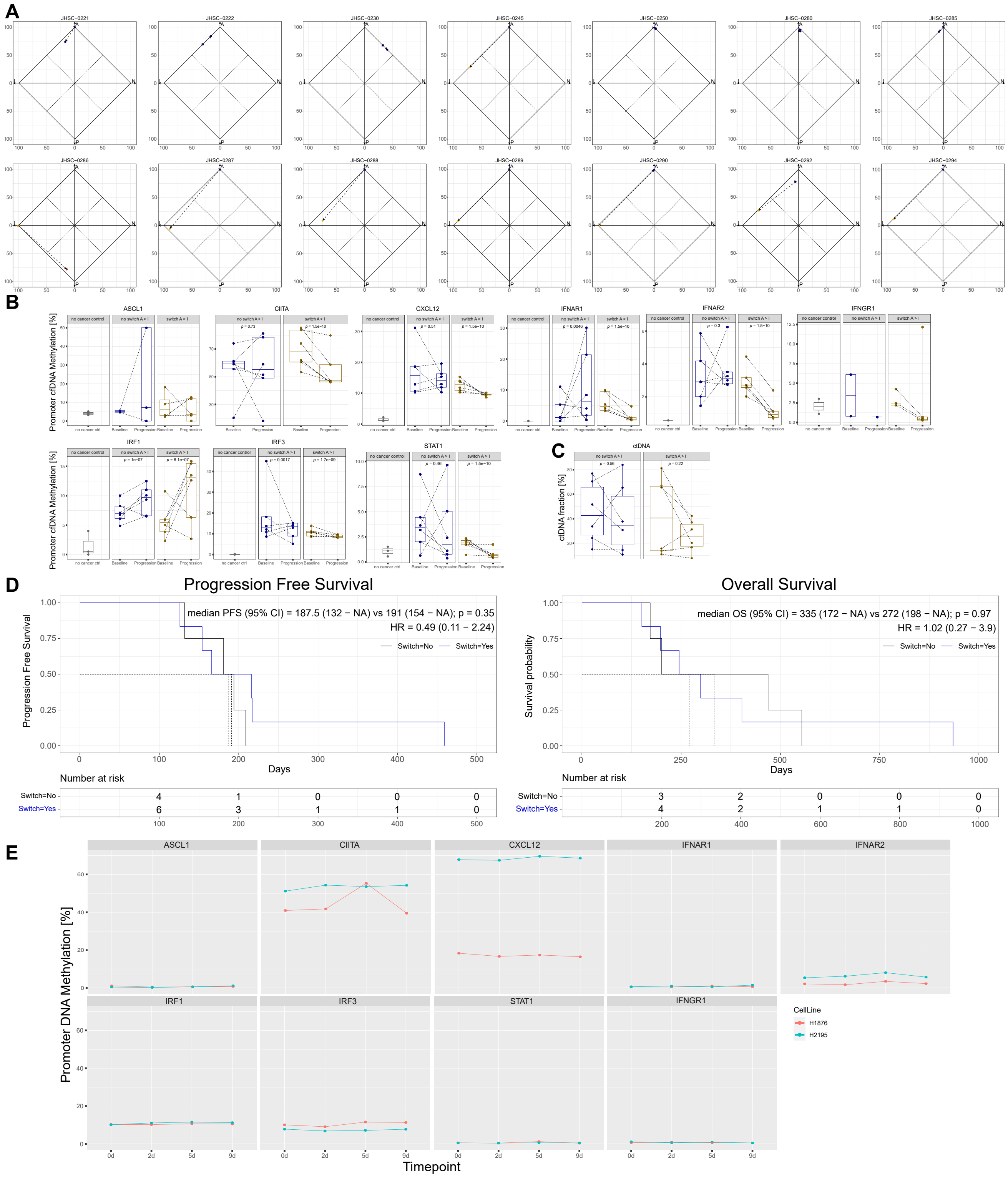

Figure S9: Longitudinal comparison of SCLC samples and analyssis of subtype switching from plasma. Related to Figure 3. **A** Characterization of SCLC consensus heterogeneity from baseline to progression in matched plasma samples. The consensus agreement value for each subtype is plotted from the subtyping at baseline and at progression demonstrating a subtype switch in a subset of samples. **B** Promoter methylation of immune-related genes in plasma samples with matched baseline and progression samples. Samples with a baseline SCLC-A subtype are compared between those who switched to a SCLC-I subtype and those who maintained the SCLC-A subtype. Wilcoxon test is used to compute significance of differences within each group. In samples where no p-value is given, the total number of comparisons was too low to allow statistical analysis but data is plotted for visual representation. * CIITA: Data on promoter region of CIITA was not of sufficient quality in the 'no cancer ctrl' samples and is thus not reported. **C** Comparison of ctDNA fraction between patients who switch from A → I and those who do not. **D** Comparison of progression free survival (PFS) and overall survival (OS) between patients whose tumor switched from SCLC-A to SCLC-I and patients whose tumor maintained the SCLC-A subtype. Hazard ratio (HR) is calculated using cox proportional hazard ration and survival differences are computed using log-rank test. Changes of promoter methylation in immune related genes in two SCLC cell lines (H1876 and H2195; both SCLC-A) with 2µM cisplatin for 9 days. Boxplot shows the median as thick line, the box highlighting the first and third quartile with the whiskers highlighting 1.5x the interquartile range.
